# Fingertip-Measured Skin Carotenoids and Advanced Glycation End Product Levels in Glaucoma

**DOI:** 10.3390/antiox11061138

**Published:** 2022-06-09

**Authors:** Yoichi Kadoh, Yuji Takayanagi, Junichi Sasaki, Masaki Tanito

**Affiliations:** Department of Ophthalmology, Shimane University Faculty of Medicine, Izumo 693-8501, Japan; m171205@med.shimane-u.ac.jp (Y.K.); y.takayanagi1008@med.shimane-u.ac.jp (Y.T.); m151047@med.shimane-u.ac.jp (J.S.)

**Keywords:** skin carotenoid, fingertip sensor, Veggie Meter, advanced glycation end products (AGEs), AGE sensor, reactive oxygen species, oxidative stress, antioxidants, primary open-angle glaucoma, exfoliation glaucoma

## Abstract

Carotenoids have antioxidant properties, and the accumulation of advanced glycation end products (AGEs) is associated with reactive oxygen species production; they have attracted attention as factors predictive of the onset and progression in glaucoma. Fingertip measurement is applicable for carotenoids and AGEs due to its noninvasiveness and simplicity. The study included 663 eyes of 663 Japanese subjects (357 males, 306 females). The mean age was 69.9 years with a standard deviation of 11.0. The study population comprised participants with primary open-angle glaucoma (PG) (*n* = 358), exfoliation glaucoma (EG) (*n* = 168), and controls (*n* = 137). Multivariate models suggested that lower skin carotenoid (SC) levels were associated with male gender (standard β = −0.14), AGE scores (−0.24), and a history of intraocular surgery (−0.22). Higher SC levels were associated with higher vegetable intake scores (0.21 for score 3) and diabetes (0.10). However, no association was seen between SCs and glaucoma type. AGEs levels were negatively associated with carotenoid scores (−0.25), PG (−0.15), and smoking habits (−0.26) and positively correlated with EG (0.14). SCs and AGEs were negatively correlated in the single regression analysis (*r* = −0.20, *p* < 0.0001). In conclusion, higher levels of AGEs may be candidates for systemic biomarkers of glaucoma associated with the exfoliation syndrome. SC levels can reflect self-reported daily vegetable intake.

## 1. Introduction

Glaucoma is a group of ophthalmic neurodegenerative diseases, and its progression is irreversible [1,2]. More than 70 million people in the world have glaucoma, making it the leading cause of low vision and blindness [3,4]. Elevated intraocular pressure (IOP) is a major risk factor, and lowering the IOP through medication or surgery is the main therapeutic option. Numerous risk factors, such as genetics, inflammation, ocular blood flow, and oxidative stress have been proposed by diverse nonclinical and clinical studies; however, the only reliable parameter used in clinical practice is IOP [5,6]. Elevated IOP levels are associated with retinal ganglion cell (RGC) death and inhibit blood flow, leading to oxidative stress [7,8,9]. Oxidative stress increases with the overproduction of reactive oxygen species (ROS) and dysfunction of the antioxidant system. Dysfunction of the mitochondria in glaucoma enhances ROS production and causes inflammatory injury of the RGCs [10,11]. Antioxidants suppress inflammation caused by ROS and improve RGCs in experimental glaucoma [12]. Neurodegeneration caused by ROS is considered a modifiable factor of glaucoma onset and progression [13,14]. ROS is mainly generated by the metabolism of the mitochondria and neutralized by antioxidants [15]. We previously reported that lower levels of serum biologic antioxidant potential were correlated with higher levels of IOP and with greater visual field loss in primary open-angle glaucoma (PG) [16,17,18].

Carotenoids, such as lutein and zeaxanthin, exist in the retina, especially in the fovea, and they are antioxidants [19,20,21,22]. These carotenoids scavenge free radicals to reduce oxidative stress and absorb visible light to inhibit the generation of light-induced ROS. Because humans cannot produce carotenoids in the body, they must be ingested [23]. The prevalence of glaucoma was lower in groups with high vegetable/fruit intake in epidemiologic studies [24,25]. Several clinical trials have been conducted to identify the neuroprotective benefits of carotenoids in glaucoma [26]. Macular pigment optical density (MPOD), which is the density of carotenoids, is lower in patients with glaucoma [27], and lutein supplementation increased carotenoid levels in the macular pigment [28,29,30,31]. Studies of dietary carotenoid supplementation in randomized controlled trials of glaucoma have been conducted; however, no significant effects were observed [32,33,34]. Even though carotenoids are supposed to play an important role in glaucoma, accurate monitoring of carotenoids is not yet possible. Percutaneous fingertip measurements may be suitable for monitoring carotenoids multiple times. These methods are noninvasive and convenient because they do not require blood sampling or difficult measurement procedures. The subjects place their fingers on the measurement device for only about 10 s for the measurement. The skin carotenoid (SC) levels obtained using the pressure-mediated reflection spectroscopy (RS) method were correlated with serum carotenoid levels measured by high-performance liquid chromatography (HPLC) [35]. The instrument can measure in the 350–850 nm range, which includes the absorption wavelengths of carotenoids around 480 nm. The association between skin melanin content and carotenoids is weakly correlated, indicating that SC levels are not affected by melanin absorption [35,36].

AGEs are generated by nonenzymatic glycation of proteins, nucleic acids, and lipids (Maillard reaction), followed by rearrangements and oxidative steps [37,38]. AGEs accumulate in ocular tissue and enhance ROS generation via the receptor for AGEs (RAGE). In the glaucomatous retina and optic nerve, the accumulation of AGEs is increased, and the expression of RAGE is upregulated. Upregulation of RAGE might cause early cell death of the RCGs [39]. AGEs levels were also measured percutaneously by the previously reported procedure using fingertip-measured skin autofluorescence (sAF) [40]. Among the AGEs, Nε-(carboxymethyl)-lysine (CML) was considered to be strongly involved in the pathogenic role, but it was difficult to detect directly because of the nonfluorescent feature of CML. sAF was correlated not only with fluorescent AGEs (Nδ-(5-hydro-5-methyl-4-imidazolone-2-yl)-ornithine, pentosidine, and collagen-linked fluorescence) but also with non-fluorescent AGEs (CML) [41,42]. This suggested that fluorescent and nonfluorescent AGEs had similar distributions, and that sAF can be used as a marker of AGE accumulation [41,42].

As part of our ongoing research program directed toward the analysis of the relationship between ophthalmologic disorders and stress markers measured by fingertip measurements [16,17,18], we investigated the clinical factors including SC and AGEs levels with glaucoma types. We previously reported AGEs levels in PG and EG [40]; however, the relationship between SC levels and glaucoma has not been reported. Herein, we report a comparison of the SC and AGEs levels in patients with and without glaucoma.

## 2. Materials and Methods

### 2.1. Subjects

The current study adhered to the tenets of the Declaration of Helsinki. The institutional review board (IRB) of Shimane University Hospital approved the research (No. 20200228-2, issued on 21 June 2021). The IRB approval did not require that each patient provide written informed consent for publication; instead, the study protocol was posted at the study institutions to opt the participants out of the study. Subjects were recruited consecutively at the Department of Ophthalmology, Shimane University Hospital from November 2019 to January 2021. This study included 663 eyes of 663 Japanese subjects in total (357 males, 306 females). The mean age was 69.9 years with a standard deviation of 11.0. The study population comprised participants with PG (*n* = 358), EG (*n* = 168), and controls (*n* = 137). Each participant underwent measurement of the best-corrected visual acuity (BCVA), Goldmann applanation tonometer-measured IOP, and slit-lamp and funduscopic examinations. The highest IOP recorded, the lens status, and the number of glaucoma medications used were collected from the previous medical charts. A combination medication was counted as two drugs. Information about the current smoking status, amount of vegetable intake, and history of diabetes and systemic hypertension was obtained during the medical interview. The amount of vegetable intake was estimated by the forced choice scale using a four-point rating system in which 0 indicated no or rarely, 1 indicated sometimes/small amount, 2 indicated frequent/sufficient amount, and 3 indicated very frequent/large amount. The diagnosis of PG was determined by the following observations: iridocorneal angles open in both eyes, distinctive appearance of glaucomatous optic neuropathy including the optic disc cup enlargement or the focal neuroretinal rim thinning, visual field defects corresponding to the optic disc appearance detected in at least in one eye, and no manifestation of secondary glaucoma seen bilaterally. The diagnosis of EG was determined on the basis of an open iridocorneal angle and distinctive pseudo-exfoliation material deposition on the lens capsule and/or pupillary margin in one or both eyes. When both eyes met the criteria, the eye with the worse visual field mean deviation (MD) was included in the PG or EG evaluation. Visual field defects were determined by the automatic visual field tester (Humphrey Visual Field Analyzer, Carl Zeiss Meditec, Dublin, CA, USA). The control subjects had no remarkable ocular disorders other than age-related cataracts, clinical findings of glaucoma, and glaucoma medication use. The highest IOP recorded was <21 mmHg in the control subjects. For controls, the eye with the better BCVA was included in the study. If the BCVA was the same in both eyes, the right eye was included. For all groups in this study, eyes with ocular pathologies other than glaucoma and age-related cataract were excluded. Patients with diabetic retinopathy were carefully excluded because a close association of AGEs with diabetes or its complications has been reported [43,44,45].

### 2.2. Measurement of Carotenoids in the Fingertip Skin

SC levels were measured by pressure-mediated RS (Veggie Meter^®^, Longevity Link Corporation, Salt Lake City, UT, USA). The Veggie Meter adopted pressure-mediated RS using a white light-emitting diode (350–850 nm) [35]. Experienced examiners performed all measurements. The measured scores were expressed as optical density (OD) units. The calibration was performed with the manufacturer-provided reference materials before the start of the morning and afternoon sessions. Participants inserted the left middle finger into the device’s finger cradle to measure the SC. The SC index was determined as the average of two consecutive measurements in 657 participants and by three measurements six 6 participants.

### 2.3. Measurement of AGEs in the Fingertip Skin

AGEs levels were measured by the AGEs Sensor (Air Water Biodesign Inc., Kobe, Japan). The sAF values were obtained at the excitation wavelength (365 nm) and emission wavelength (440 nm). Experienced examiners performed all measurements. The measured sAF was expressed as the AGE index in arbitrary units (AU). The AGE index was determined as the average of two consecutive measurements in 641 participants and by three measurements in 21 participants. For the triple AGE measurements, the mean coefficient of variation and Cronbach’s α intraclass correlation coefficient were 6.7% ± 7.3% and 0.938, respectively, in our pilot study.

### 2.4. Statistical Analysis

The data were expressed as numbers and percentages for categorical parameters, and as mean ± standard deviation (SD) with 95% confidence interval (CI) ranges for continuous parameters. The decimal BCVA was converted into the logarithm of the minimum angle of resolution (logMAR). Respective counting fingers, hand motions, light perception, and no light perception values were considered as the decimal visual acuities of 0.0025, 0.002, 0.0016, and 0.0013 [46]. For categorical parameters, group comparisons were performed using the G-test followed by Fisher’s exact probability test. For continuous parameters, group comparisons were performed by one-way analysis of variance (ANOVA) followed by unpaired *t*-tests. To correct for multigroup comparisons, using Bonferroni’s correlation, *p*-values of 0.0167 and 0.0033 were regarded as the significance levels of 5% and 1%, respectively, for the Fisher’s exact probability test or unpaired *t*-tests. Possible correlations among AGEs, SCs, and other parameters were calculated by the unpaired *t*-test for categorical variables, and by linear regression analyses with Pearson’s correlation coefficient for continuous variables. To correct for vegetable intake score, which has four categories, *p*-values of 0.0083 and 0.0016 for the Fisher’s exact probability test were regarded as the significance levels of 5% and 1%, respectively. We conducted further multiple regression analyses for possible associations among AGEs and SCs with assorted parameters to adjust differences among groups. JMP Pro statistical software version 16.1.0 (SAS Institute, Inc., Cary, NC, USA) was used for all statistical calculations in this study.

## 3. Results

The demographic characteristics of the patients, i.e., age, sex, BCVA, IOP, highest IOP, number of glaucoma medications, MD, lens status, current smoking status, diabetes, hypertension, vegetable intake scores, history of intraocular surgery, AGE scores, and SC scores, are shown in Table 1. Sex, current smoking status, diabetes, vegetable intake score, and carotenoids did not differ among the three groups, while the other parameters including AGE scores differed. The SC scores did not differ significantly among the three groups. However, the AGE scores of EG (0.48 ± 0.10) were significantly higher than those of the PG (0.44 ± 0.08, *p* < 0.0001) and control group (0.45 ± 0.08, *p* = 0.0012). No significant difference was seen between the PG and control group. The data underlying this article was described in Table S1.

According to univariate analysis, no parameters of the continuous variables were correlated with the SC scores (Table 2). Male (*p* < 0.0001) and current smoking status (*p* < 0.0001) were associated with lower SC levels than their corresponding group (Table 3).

Age (*p* < 0.0001) and BCVA (*p* < 0.0001) were positively correlated with AGE scores in the univariate regression analysis of continuous variables, whereas the IOP, highest IOP, and number of glaucoma medications were not correlated with AGE scores (Table 4). PG (*p* < 0.0001) and current smoking status (*p* < 0.0001) were associated with lower AGE levels than their corresponding group (Table 5).

Table 6 shows the association between vegetable intake scores with AGEs scores and SC scores. SC scores differed depending on vegetable intake scores. The SC score of vegetable intake score 3 group (393 ± 124) was significantly higher than that of the score 0 (282 ± 112, *p* < 0.0001), 1 (267 ± 94, *p* < 0.0001), and 2 (323 ± 118, *p* < 0.0001) groups. The SC score of vegetable intake score 2 group was also significantly higher than that of the score 0 (*p* = 0.047) and 1 (*p* < 0.0001) groups. There was no significant difference in SC scores between vegetable intake score 1 and 0 groups. The AGE scores did not differ among the vegetable intake score groups.

The multiple regression model analysis of SCs scores and various parameters is shown in Table 7. Male (female, standard β = −0.14, *p* = 0.0045), AGE scores (AU, standard β = −0.24, *p* < 0.0001), and a history of intraocular surgery (no, standard β = −0.22, *p* = 0.0359) were negatively correlated with lower SC scores, while diabetes (no, standard β = 0.10, *p* = 0.0376) was positively correlated with higher SC scores (Table 7). A vegetable intake score of 3 was correlated with higher SC scores (0, standard β = 0.21, *p* < 0.0001); however, the vegetable intake score 1 was correlated with lower SC scores (0, standard β = −0.16, *p* = 0.0014).

The multiple regression model analysis of the AGE scores and various parameters is shown in Table 8. SC scores (OD, standard β = −0.25, *p* < 0.0001), PG (control, standard β = −0.15, *p* = 0.0112), and smoking status (no, standard β = −0.26, *p* < 0.0001) were negatively correlated with the AGE scores level, while EG (control, standard β = 0.14, *p* = 0.0173) was positively correlated with the AGE scores level.

**Figure 1:**
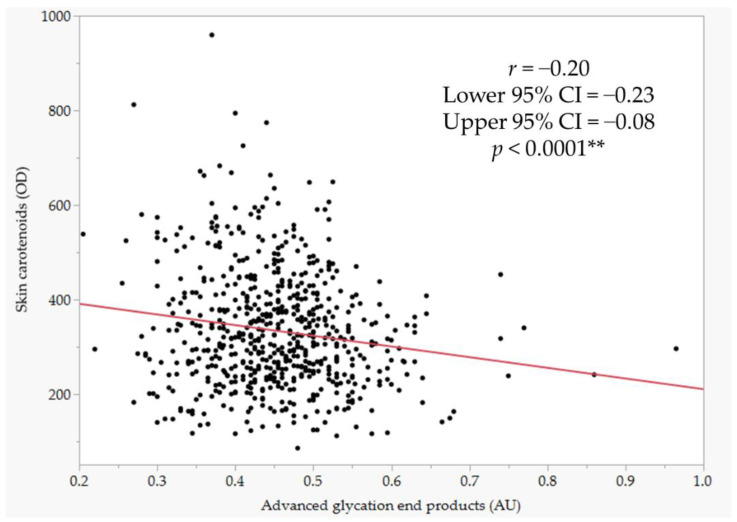
Single regression of AGEs and SCs. The *p*-value was estimated by a linear regression analysis. Significance level at 1% (*p* < 0.01) **.

The scatterplot in Figure 1 shows the correlation between AGE scores and SC scores. According to regression analysis, they were significantly correlated with each other. The correlation coefficient (*r*) was −0.20, and the *p*-value was less than 0.0001.

## 4. Discussion

To the best of our knowledge, this study is the first to simultaneously estimate the AGE and SC levels using fingertip sensors in patients with glaucoma. This study found that low SC levels were associated with male gender, history of intraocular surgery, current smoking status, diabetes, low vegetable intake score, and high levels of AGEs by multiple regression analysis; however, no significant association with glaucoma type was detected. On the other hand, AGEs levels were higher in EG than PG and controls. In addition, a negative association was found between SC and AGEs levels.

SC levels measured by the Veggie Meter^®^ were strongly correlated with serum carotenoid levels detected by HPLC [35]. The meter, using pressure-mediated RS, detects skin chromophores between 400 and 750 nm. Most carotenoids, such as α- and β-carotenes, β-cryptoxanthin, lycopene, lutein, and zeaxanthin, have a maximal absorption wavelength of around 480 nm; therefore, the carotenoid score reflects the bulk of these carotenoid molecules. Intraocular levels of lutein, (3*R*,3′*R*)-zeaxanthin, and *meso*-(3*R*,3′*S*)-zeaxanthin, the only carotenoids present in macular pigment [22], and their antioxidant activity are difficult to estimate directly. Although the carotenoid levels reflect previous intake of vegetables, this lifestyle factor is also difficult to determine. Given that SC levels were mainly associated with vegetable intake for about 1 month [47], SC can be a good endpoint to assess the roles of carotenoids in various diseases in clinical situations. In this study, the role of SCs in the differentiation of glaucoma types or the suitability of SCs in glaucoma management was not determined. Ophthalmic neurodegeneration in glaucoma occurs over several decades [1,4]; therefore, the risk factors for disease onset and progression should be affected by time course. Fluctuation in each patient’s vegetable intake over a long period of years would be one explanation for the absence of a correlation between SC and glaucoma. Because of the bulked estimation of various carotenoids, the roles of specific carotenoid molecules might be masked in our methodology. Several factors were correlated with SCs by multiple regression analysis (Table 7). The results for smoking status [48] and gender [23] were consistent with previous reports, suggesting the proper estimation of carotenoid levels by this study. Several epidemiologic studies reported that the group with higher fruit/vegetable intake had a lower prevalence of glaucoma [24]. Administration of carotenoid-containing supplements to healthy subjects increased carotenoid levels in serum and MPOD [29,31,49]. Serum levels of carotenoids have not been evaluated in previous epidemiologic and carotenoid administration studies in glaucoma, although MPOD has been measured [26]. The literature did not contain any relevant previous studies that assessed the association between SC levels and the type of glaucoma (PG, EG). Fingertip-measured carotenoid levels would be beneficial for use in future studies due to its compatibility.

Significant differences in AGEs were observed by glaucoma type, with higher AGEs in EG and lower AGEs in PG (Table 8). The result of AGEs in EG was consistent with our previous report [40]. Exfoliation products are produced by the aggregation of microfibrils [50], and the effect of AGEs via RAGE may enhance the production. Tezel et al. reported that AGE deposition and RAGE upregulation were observed in the retina and optic nerve head in glaucoma. The RAGE upregulation in RGCs and glia might be involved in early optic nerve degeneration [39]. Since the production of AGEs enhanced by ROS was irreversible and this accumulation proceeded over years, short-term fluctuations were small; therefore, AGEs measured by fingertip would reflect AGEs in the eye [37]. The reason why AGEs were lower in PG was unclear but suggested that the background factors of the disease differed from those of EG. SCs and AGEs were negatively correlated with each other (Figure 1). Dietary intake of carotenoids would be involved in modifying AGE accumulation in the body. The formation process of AGEs includes oxidative reactions [38]; therefore, the antioxidant effect of carotenoids suppressed these oxidation steps to reduce the production of AGEs accordingly [13]. This was supported by a report that administration of chestnut (*Tarpa bispinosa* Roxb.) extract and lutein decreased the AGEs levels [51].

The current study had several limitations. The subject demographic data (Table 1) differed significantly in age, BCVA, IOP, highest IOP, number of glaucoma medications, MD, phakia status, hypertension, and history of intraocular surgery. These factors might have affected the results, although we attempted to minimize the effect using multivariate analyses (Table 7 and Table 8). Current smoking habits, diabetes, hypertension, and vegetable intake scores were collected by interviews, which might lower the power of detection. Although the vegetable intake score was not obtained using a standard questionnaire [52], our method would be beneficial because it was correlated with SC levels. Since the Veggie Meter^®^ detects carotenoids as mixtures, it might not be able to extract the specific carotenoids associated with glaucoma pathotypes. Long-term measurements and more accurate methods are desirable to elucidate the relationship between carotenoid levels measured by fingertip and glaucoma. Given its easy-to-measure nature, estimation of both SCs and AGEs by fingertip can be applicable for exploring the biomarkers of various pathologies other than eye diseases.

## 5. Conclusions

In conclusion, this study did not detect an association between SC levels and glaucoma types, although SC levels were associated with vegetable intake scores. Furthermore, higher levels of AGEs may be useful candidates as systemic biomarkers of glaucoma associated with exfoliation syndrome; AGEs might be useful to distinguish two types of open-angle glaucoma.

## Figures and Tables

**Table 1:** Demographic subject data.

Group	Control	PG	EG	*p*-Value ^a^
	137	358	168	
Age (years)				
*n*	137	358	168	
Mean ± SD	73.4 ± 10.9	66.1 ± 10.7	75.1 ± 8.6	<0.0001 **
95% CI	71.5 to 75.2	65.0 to 67.2	73.7 to 76.3	
		vs. control, *p* < 0.0001 ^b^ ##	vs. control, *p* = 0.1322 ^b^	
			vs. PG, *p* < 0.0001 ^b^ ##	
Sex				
Male, *n* (%)	64 (47)	203 (57)	90 (54)	0.1364
Female, *n* (%)	73 (53)	155 (43)	78 (46)	
BCVA (logMAR)				
*n*	137	358	168	
Mean ± SD	0.16 ± 0.22	0.19 ± 0.42	0.35 ± 0.61	0.0003 **
95% CI	0.13 to 0.20	0.15 to 0.24	0.25 to 0.44	
		vs. control, *p* = 0.4680 ^b^	vs. control, *p* = 0.0010 ^b^ ##	
			vs. PG, *p* = 0.0008 ^b^ ##	
IOP (mmHg)				
*n*	90	357	168	
Mean ± SD	15.0 ± 2.7	15.4 ± 7.0	18.0 ± 9.2	0.0004 **
95% CI	14.4 to 15.6	14.7 to 16.2	16.6 to 19.4	
		vs. control, *p* = 0.5504 ^b^	vs. control, *p* = 0.0030 ^b^ ##	
			vs. PG, *p* = 0.0006 ^b^ ##	
Highest IOP (mmHg)				
	92	358	168	
Mean ± SD	15.4 ± 3.5	21.4 ± 8.6	27.1 ± 11.4	<0.0001 **
95% CI	14.6 to 16.1	20.5 to 22.3	25.4 to 28.9	
		vs. control, *p* < 0.0001 ^b^ ##	vs. control, *p* < 0.0001 ^b^ ##	
			vs. PG, *p* < 0.0001 ^b^ ##	
No. of glaucoma medications				
*n*	137	358	168	
Mean ± SD	0	2.5 ± 1.3	2.5 ± 1.4	< 0.0001 **
95% CI		2.4 to 2.7	2.3 to 2.7	
		vs. control, *p* < 0.0001 ^b^ ##	vs. control, *p* < 0.0001^b^ ##	
			vs. PG, *p* = 0.8741^b^	
MD (dB)				
*n*		358	168	
Mean ± SD	–	−15.9 ± 8.4	−18.0 ± 9.8	0.0114 **
95% CI		−16.8 to −15.0	−19.4 to –16.7	
Pseudophakia				
Yes, *n* (%)	19 (14)	179 (50)	119 (71)	<0.0001 **
No, *n* (%)	118 (86)	179 (50)	49 (29)	
		vs. control, *p* < 0.0001 ^b^ ##	vs. control, *p* < 0.0001 ^b^ ##	
			vs. PG, *p* < 0.0001 ^b^ ##	
Current smoking				
Yes, *n* (%)	15 (11)	41 (12)	18 (11)	0.9586
No, *n* (%)	122 (89)	315 (88)	150 (89)	
Diabetes				
Yes, *n* (%)	21 (21)	53 (23)	26 (20)	0.8220
No, *n* (%)	78 (79)	177 (77)	102 (80)	
Hypertension				
Yes, *n* (%)	48 (49)	133 (55)	85 (65)	0.0449 *
No, *n* (%)	49 (51)	107 (45)	45 (35)	
		vs. control, *p* = 0.3366 ^b^	vs. control, *p* = 0.0205 ^b^	
			vs. PG, *p* = 0.0764 ^b^	
Vegetable intake score				
*n*	134	349	167	
0, *n* (%)	11 (8)	25 (7)	16 (10)	0.8103
1, *n* (%)	23 (17)	48 (14)	28 (17)	
2, *n* (%)	72 (54)	191 (55)	89 (53)	
3, *n* (%)	28 (21)	85 (24)	34 (20)	
Intraocular surgery				
Yes, *n* (%)	19 (14)	223 (62)	122 (73)	<0.0001 **
No, *n* (%)	118 (86)	135 (38)	46 (27)	
		vs. control, *p* < 0.0001 ^b^ ##	vs. control, *p* < 0.0001 ^b^ ##	
			vs. PG, *p* = 0.0235 ^b^	
SCs (OD)				
*n*	137	358	168	
Mean ± SD	327.8 ± 125.7	336.2 ± 125.6	330.6 ± 114.0	0.7974
95% CI	306.6 to 348.9	323.2 to 349.3	313.2 to 347.9	
AGEs (AU)				
*n*	137	358	168	
Mean ± SD	0.45 ± 0.08	0.44 ± 0.08	0.48 ± 0.10	<0.0001 **
95% CI	0.43 to 0.46	0.43 to 0.45	0.46 to 0.49	
		vs. control, *p* = 0.6818 ^b^	vs. control, *p* = 0.0012 ^b^ ##	
			vs. PG, *p* < 0.0001 ^b^ ##	

^a^ *p*-Values were estimated by ANOVA for continuous variables and by G-test for categorical variables. ^b^ Post hoc tests were performed by *t*-test or Fisher’s exact probability test. Significance levels at 5% (*p* < 0.05) *, 1% (*p* < 0.01) **, and 1% (*p* < 0.0033) ##. PG, primary open-angle glaucoma; EG, exfoliation glaucoma.

**Table 2:** Possible associations among SCs and various continuous parameters.

Parameters	*r*	Lower 95% CI	Upper 95% CI	*p*-Value
Age (years)	0.03	−0.05	0.10	0.4966
BCVA (logMAR)	−0.06	−0.13	0.02	0.1359
IOP (mmHg)	0.03	−0.05	0.11	0.4243
Highest IOP (mmHg)	−0.02	−0.10	0.06	0.6725
No. of glaucoma medications	0.06	−0.02	0.14	0.1200

Pearson’s correlation coefficient (*r*).

**Table 3:** Possible association among SCs and various categorical parameters.

Parameters	Mean ± SD (95% CI)	Mean ± SD (95% CI)	*p*-Value
Sex	Male, 310 ± 122 (297 to 323)	Female, 360 ± 118 (347 to 374)	<0.0001 **
Pseudophakia	Yes, 332 ± 122 (319 to 346)	No, 334 ± 123 (321 to 347)	0.8133
Glaucoma type	PG, 336 ± 126 (323 to 349)	EG, 331 ± 114 (313 to 348)	0.6210
Intraocular surgery	Yes, 340 ± 123 (326 to 353)	No, 328 ± 123 (326 to 353)	0.2360
Current smoking	Yes, 252 ± 84 (232 to 271)	No, 344 ± 123 (333 to 354)	<0.0001 **
Diabetes	Yes, 342 ± 136 (315 to 369)	No, 330 ± 119 (318 to 343)	0.4285
Hypertension	Yes, 325 ± 128 (309 to 340)	No, 341 ± 118 (325 to 358)	0.1284

The *p*-values were estimated by *t*-test between groups. Significance level at 1% (*p* < 0.01) **.

**Table 4:** Possible associations among AGEs and various continuous parameters.

Parameters	*r*	Lower 95% CI	Upper 95% CI	*p*-Value
Age (year)	0.17	0.10	0.24	<0.0001 **
BCVA (logMAR)	0.15	0.08	0.23	<0.0001 **
IOP (mmHg)	0.03	−0.05	0.10	0.5333
Highest IOP (mmHg)	0.00	−0.08	0.08	0.9929
No. of glaucoma medications	0.01	−0.07	0.08	0.8753

Pearson’s correlation coefficient (*r*). Significance level at 1% (*p* < 0.01) **.

**Table 5:** Possible association among AGEs and various categorical parameters.

Parameters	Mean ± SD (95% CI)	Mean ± SD (95% CI)	*p*-Value
Sex	Male, 0.46 ± 0.09 (0.45 to 0.47)	Female, 0.45 ± 0.09 (0.44 to 0.46)	0.1209
Pseudophakia	Yes, 0.46 ± 0.09 (0.45 to 0.47)	No, 0.45 ± 0.08 (0.44 to 0.46)	0.3183
Glaucoma type	PG, 0.44 ± 0.08 (0.43 to 0.45)	EG, 0.48 ± 0.10 (0.46 to 0.49)	<0.0001 **
Intraocular surgery	Yes, 0.46 ± 0.09 (0.45 to 0.47)	No, 0.45 ± 0.08 (0.44 to 0.46)	0.2130
Current smoking	Yes, 0.40 ± 0.09 (0.39 to 0.42)	No, 0.46 ± 0.08 (0.45 to 0.47)	<0.0001 **
Diabetes	Yes, 0.46 ± 0.09 (0.45 to 0.48)	No, 0.45 ± 0.09 (0.44 to 0.46)	0.2648
Hypertension	Yes, 0.46 ± 0.09 (0.45 to 0.47)	No, 0.45 ± 0.08 (0.44 to 0.46)	0.1284

The *p*-values were estimated by *t*-test between groups. Significance level at 1% (*p* < 0.01) **.

**Table 6:** Possible association between vegetable intake scores with SCs and AGEs.

	Vegetable Intake Score				
	0	1	2	3	
*n*	52	99	352	147	
**Parameters**	**Mean ± SD** **(95% CI)**	**Mean ± SD** **(95% CI)**	**Mean ± SD** **(95% CI)**	**Mean ± SD** **(95% CI)**	***p*-Value ^a^**
SCs (OD)	282 ± 112 (255 to 317)	267 ± 94 (249 to 286)	335 ± 118 (323 to 347)	393 ± 124 (373 to 413)	<0.0001 **
		vs. 0, *p* = 0.1731 ^b^	vs. 0, *p* = 0.0047 ^b^ #	vs. 0, *p* < 0.0001 ^b^ ##	
			vs. 1, *p* < 0.0001 ^b^ ##	vs. 1, *p* < 0.0001 ^b^ ##	
				vs. 2, *p* < 0.0001 ^b^ ##	
AGEs (AU)	0.47 ± 0.10 (0.45 to 0.50)	0.45 ± 0.09 (0.44 to 0.47)	0.45 ± 0.09 (0.45 to 0.46)	0.44 ± 0.08 (0.43 to 0.46)	0.1956

^a^ *p*-Values were estimated by G-test. ^b^ Post hoc comparisons were performed by Fisher’s exact probability test. Significance levels at 1% (*p* < 0.01) **, 5% (*p* < 0.0083) #, and 1% (*p* < 0.0016) ##.

**Table 7:** Possible associations among SCs and various parameters analyzed by multiple regression analysis.

Parameters	Estimate	Lower 95% CI	Upper 95% CI	*p* Value	Standard β
Age (year)	0.64	−0.62	1.91	0.3200	0.06
Male (female)	−16.98	−28.65	−5.30	0.0045 **	−0.14
BCVA (logMAR)	−7.91	−33.16	17.33	0.5380	−0.03
IOP (mmHg)	−0.56	−2.63	1.52	0.5984	−0.04
Highest IOP (mmHg)	0.80	−1.12	2.72	0.4121	0.06
No. of glaucoma medications	6.70	−2.96	16.38	0.1733	0.09
AGEs (AU)	−346.64	−484.02	−209.26	<0.0001 **	−0.24
Phakia (pseudophakia)	−17.92	−43.48	7.63	0.1686	−0.14
Intraocular surgery, yes (no)	−27.17	−52.54	−1.79	0.0359 *	−0.22
PG (control)	−4.08	−23.08	14.93	0.6734	−0.02
EG (control)	9.98	−11.09	31.05	0.3524	0.05
Current smoking, yes (no)	−41.72	−61.15	−22.30	<0.0001 **	−0.21
Diabetes, yes (no)	15.79	0.91	30.67	0.0376 *	0.10
Hypertension, yes (no)	−7.97	−19.73	3.79	0.1837	−0.06
Vegetable intake score 1 (0)	−41.04	−66.06	−16.03	0.0014 **	−0.16
Vegetable intake score 2 (0)	10.60	−7.17	28.36	0.2415	0.05
Vegetable intake score 3 (0)	48.79	26.75	70.83	<0.0001 **	0.21

*p*-Values were estimated by a multiple regression model. Significance levels at 5% (*p* < 0.05) * and 1% (*p* < 0.01) **.

**Table 8:** Possible associations among AGEs and various parameters analyzed by multiple regression analysis.

Parameters	Estimate	Lower 95% CI	Upper 95% CI	*p*-Value	Standard β
Age (year)	0.001	−0.00009	0.002	0.0784	0.10
Male (female)	0.078	−0.0005	0.016	0.0661	0.09
BCVA (logMAR)	0.003	−0.015	0.021	0.7230	0.02
IOP (mmHg)	−0.002	−0.002	0.013	0.8295	−0.02
Highest IOP (mmHg)	−0.003	−0.002	0.001	0.6913	−0.03
No. of glaucoma medications	0.057	−0.001	0.013	0.1040	0.10
Phakia (pseudophakia)	0.019	0.0008	0.037	0.2203	0.22
Intraocular surgery, yes (no)	0.014	−0.004	0.032	0.1315	0.16
SCs (OD)	−0.0002	−0.0002	−0.001	<0.0001 **	−0.25
PG (control)	−0.018	−0.031	−0.004	0.0112 *	−0.15
EG (control)	0.018	0.003	0.033	0.0173 *	0.14
Current smoking, yes (no)	−0.036	−0.050	−0.022	<0.0001 **	−0.26
Diabetes, yes (no)	0.008	−0.002	0.019	0.1237	0.08
Hypertension, yes (no)	0.004	−0.005	0.012	0.4099	0.04
Vegetable intake score 1 (0)	−0.006	−0.024	0.012	0.5120	−0.03
Vegetable intake score 2 (0)	−0.008	−0.021	0.005	0.2181	−0.06
Vegetable intake score 3 (0)	−0.005	−0.021	0.012	0.5814	−0.03

*p*-Values were estimated by a multiple regression model. Significance levels at 5% (*p* < 0.05) * and 1% (*p* < 0.01) **.

## Data Availability

The data are contained within the article and Supplementary Materials.

## Supplementary Materials

The following supporting information can be downloaded at: https://www.mdpi.com/article/10.3390/antiox11061138/s1, Table S1: Data underlying the manuscript.
