# Supplementary material for: Fingertip-Measured Skin Carotenoids and Advanced Glycation End Product Levels in Glaucoma
Source: Antioxidants (Basel). 2022 Jun 9;11(6):1138. doi: 10.3390/antiox11061138 (PMC9220224; doi:10.3390/antiox11061138)
Supplement: Supplementary file 1 [file antioxidants-11-01138-s001.zip › antioxidants-1718314-supplementary.pdf]

Table S1. Data underlying the manuscript.

Study ID	Disease	Age	Sex	AGEs	Vegi	Smoke	Intake	HT	DM	LogMAR	IOP	IOPhigh	Med	MD	Lens	Surgery
1	control	72	F	0.455	224	0		y	n	0.09691	16	16	0		y	n
2	POAG	70	M	0.42	350	0		n	n	0.154902	55	55	5	-27.93	IOL	y
7	POAG	49	F	0.45	475	0		n	n	-0.07918	20	23	0	-4.43	y	n
8	POAG	39	M	0.295	338.5	0				0	10	30	3	-14.41	y	y
9	POAG	62	M	0.506667	248	0		y	n	-0.07918	21	21	4	-29.91	y	n
14	POAG	59	M	0.345	164	0		y	y	0.522879	14	14	0	-6.59	y	n
19	POAG	68	M	0.575	164.5	1	2	y	n	0	15	27	4	-9.95	y	y
22	EXG	75	F	0.5	330	0	3	y	n	0.30103	16	16	3	-18.63	y	n
23	POAG	70	F	0.295	244.5	0	2	n	n	0.045757	19	19	4	-8.15	IOL	y
24	POAG	66	F	0.86	240.5	0	2	n		1.09691	7	17	0	-19.7	IOL	y
25	POAG	73	M	0.555	130	0	2	n	n	0.154902	15	15	1	-25.94	y	y
26	EXG	70	F	0.395	539.5	0	3	n	n	0.154902	15	15	3	-27.75	y	n
27	POAG	66	M	0.43	359	1	3	n	n	-0.07918	13	13	4	-14.67	y	n
29	control	88	F	0.545	225.5	0	2	y	n	0.30103	17	17	0		y	n
39	control	65	M	0.42	212	1	2	y	n	-0.07918	21	21	0		y	n
42	EXG	71	F	0.3	541.5	0	3	n	n	-0.07918	11	11	2	-18.33	y	n
43	POAG	71	F	0.33	345	1	3	y	n	0.09691	17	17	5	-28.79	IOL	y
44	POAG	53	M	0.383333	297	0	2	n	n	-0.07918	13	13	2	-17.2	y	n
45	EXG	88	M	0.465	269	0	2	y	n	0.09691	37	37	3	-28.24	IOL	y
46	EXG	65	M	0.506667	358.5	0	3	n		-0.07918	24	30	3	-15.12	y	y
47	EXG	87	M	0.43	312.5	0	3	y	n	0.522879	8	8	3	-25.58	IOL	y
48	control	45	M	0.475	178	1	2	n	n	-0.07918	11	11	0		y	n
49	control	84	F	0.595	254	0	2	y	n	0.221849	15	15	0		y	n
52	control	85	F	0.41	325.6667	0	2	y	y	0			0		IOL	y
53	control	88	F	0.35	294.5	0	2	n	n	-0.07918	10	11	0		IOL	y
54	control	96	F	0.5	301	0	2	y	n	0.39794	15	20	0		IOL	y
55	EXG	88	M	0.575	310	0	2	n	n	0.221849	15	15	3	-24.03	y	n
59	control	66	M	0.465	521	0	2			-0.07918	14	14	0		IOL	y
60	control	70	M	0.475	548	0	3	n	n	0.221849	19	19	0		y	n
61	POAG	62	F	0.46	436	0	2	n	n	-0.07918	17	17	3	-27.12	y	n
62	POAG	77	F	0.425	442	0	3	y	y	0.09691	15	15	1	-11.24	IOL	y
63	POAG	72	M	0.5	320	0	2			0.154902	13	43	3	-28.75	IOL	y
64	POAG	20	F	0.475	227	0	2			-0.07918	36	36	0	-1.33	y	n
65	EXG	82	M	0.495	489.5	0	2	y	n	0	31	31	4	-13.56	IOL	y
66	EXG	77	F	0.386667	245.5	0	3	y	n	0.39794	16	16	2	-26.07	IOL	y
68	EXG	67	M	0.37	260	0	0	n	n	1.30103	28	28	3	-29.48	y	n
71	EXG	85	M	0.55	234.5	0	2			2.69897	12	25	3	-23.87	IOL	y
72	POAG	60	M	0.48	377	0	0	n	y	2	36	36	4	-26.36	y	n
75	control	61	F	0.445	390.5	0	2	n	n	0.09691	12	12	0		y	n
77	EXG	72	F	0.52	527	0	2	n	n	1.30103	28	28	5	-25.84	y	n
78	control	79	F	0.415	382	0	3	y	n	-0.07918	13	13	0		IOL	y
79	control	73	F	0.405	481.5	0	2	y	n	-0.07918	15	17	0		IOL	y
81	EXG	82	M	0.68	162.5	0	0	y	y	1.522879	38	38	2	-30.38	y	n
82	POAG	27	F	0.45	162.5	0	0	n	n	-0.07918	13	13	2	-12.1	y	n
85	EXG	70	F	0.505	482	0	1	y	n	0.154902	65	65	6	-7.9	IOL	y
87	EXG	77	F	0.435	272.5	0	2			0.221849	13	13	1	-21.34	IOL	y
88	POAG	76	M	0.48	299	0	2	n	n	2	7	23	0	-23	IOL	y
91	control	65	F	0.545	170	0	2	n	n	0.522879	20	20	0		y	n
92	control	70	M	0.49	456	0	0	y		0.09691	16	16	0		y	n
93	control	63	M	0.46	375	0	2	y	n	0	19	19	0		y	n
94	control	62	F	0.525	225.5	0	0	y	n	-0.07918	18	18	0		y	n
95	POAG	54	M	0.525	341	0	2	n	n	-0.07918	28	28	4	-20.9	y	n
96	POAG	67	F	0.405	549.5	0	2			0.823909	20	20	1	-7.01	y	n
97	POAG	69	M	0.52	241.5		0	n	n	0.045757	17	17	3	-22.85	y	y
98	POAG	67	F	0.42	306.5	0	2	y	n	0.045757	22	22	1	-3.22	y	n
100	POAG	58	M	0.445	410	1	3			0.69897	17	20	3	-16.24	y	y
101	POAG	80	M	0.585	437.5	0	2	y	y	0.522879	43	43	3	-9.91	y	n
102	POAG	71	M	0.51	209.5	0	3			0.154902	17	17	3	-20.39	IOL	y
103	control	48	F	0.585	226.5	0	1	n	n	-0.07918	16	16	0		y	n
104	control	79	M	0.495	476.5	0	3			0.09691			0		y	n
105	POAG	62	M	0.56	190	0	2	y	y	0.045757	15	17	3	-14.21	IOL	y
109	EXG	86	M	0.41	412.5	0	2	y	y	0.045757	14	35	0	-13.72	IOL	y
110	EXG	85	F	0.32	400	0	3	y	n	-0.07918	11	17	0	-18.94	IOL	y
111	POAG	63	M	0.435	188.5	1	2	y	y	0	21	25	3	-20.05	IOL	y
113	POAG	70	M	0.475	233	0	2	y	n	0.30103	13	16	3	-18.56	IOL	y
115	EXG	62	F	0.63	344	0	0		y	1.39794	24	24	0	-29.42	IOL	y
118	control	68	F	0.46	440.5	0	0			-0.07918	13	13	0		IOL	y
121	control	68	M	0.415	202.5	1	0	y	n	0.522879	15	15	0		y	n
122	control	48	F	0.46	164.5	1	0		n	-0.07918	17	17	0		y	n
127	EXG	87	F	0.48	325.5	0	2	y	n	0.09691	5	5	5	-23.57	IOL	y
129	POAG	81	M	0.45	267	0		y	y	0.154902	11	16	3	-16.18	y	n
130	EXG	79	M	0.415	384.5	0	2	y	n	0	27	27	3	-23.32	IOL	y
131	POAG	66	M	0.376667	555	0	0	y	y	-0.07918	21	21	4	-5.38	IOL	y
132	control	88	F	0.53	306	0	2	y	n	-0.07918	14	14	0		y	n
134	POAG	78	F	0.33	443.5	0	2	n	n	0.154902	13	20	2	-12.52	IOL	y
136	EXG	84	F	0.5	491.5	0	1	y	n	0.221849	9	11	2	-26.49	IOL	y
137	POAG	74	F	0.47	306	0	2			-0.07918	13	14	3	-9.49	IOL	y
138	POAG	72	F	0.285	285	0	1	y	n	0.69897	7	21	0	-19.69	IOL	y
142	POAG	59	F	0.45	226.5	0	1	y	y	0.09691	25	25	4	-18.38	y	n
144	POAG	60	M	0.415	406.5	0	2	n	n	0	16	16	2	-14.54	y	y
145	EXG	76	M	0.485	323	0	3	y	n	0.09691	20	20	2	-13.82	y	n
146	POAG	68	F	0.465	543	0	3	n	n	0.221849	22	22	2	-7.73	y	n
147	EXG	65	M	0.37	441.5	0	2	y	y	-0.07918	47	47	4	-16.3	IOL	y
148	POAG	76	M	0.38	682.5	0	2	n	n	0.30103	13	14	0	-3.38	y	n
150	POAG	64	M	0.22	294.5	1	2			0.39794	13	28	2	-30.48	y	y
151	control	72	F	0.48	411.5	0	3	n	y	0.045757	14	14	0		y	n
152	POAG	71	M	0.435	288	0	0	n	y	0.30103	15	16	3	-30.97	IOL	y
155	POAG	66	F	0.425	425	0	3	n	n	-0.07918	15	21	1	-11.03	IOL	y
157	control	75	F	0.465	252.5	0	2	n	n	0.30103	17	17	0		y	n
159	control	88	F	0.52	569	0	3	n	n	0.39794	18	18	0		y	n
160	control	68	F	0.455	457.5	0	2	y	n	0.221849	11	11	0		y	n
162	EXG	68	M	0.505	197.5	0	2	y	n	0.09691	28	28	4	-22.3	y	n
163	EXG	66	F	0.53	300.5	0	2	n	n	0.154902	19	19	3	-24.71	y	n
164	POAG	86	F	0.63	362.5	0	1	y	n	0.30103	6	6	3	-14.06	y	n
165	EXG	69	F	0.365	292.5	1	2			0.39794	17	24	2	-22.62	IOL	y
166	POAG	63	M	0.415	255.5	0	2	y	y	0.69897	23	23	3	-4.16	y	n

167	POAG	86	M	0.595	117.5	0	2	n	y	2.69897	41	41	4	-30.35	IOL	y
169	EXG	72	F	0.485	452	0	3	y	n	0	16	18	3	-19.5	IOL	y
175	control	74	M	0.485	353	1	2	y	y	0	19	19	0		y	n
176	control	73	F	0.475	557	0	3	y		0.221849			0		y	n
177	control	73	F	0.45	534.5	0	2	n	n	-0.07918	18	18	0		IOL	y
178	control	70	M	0.55	260.5	0	0	y	n	0	18	18	0		y	n
179	EXG	67	M	0.46	285	1	2			0	19	21	0	-1.84	y	n
182	POAG	68	M	0.615	269.5	0	2	y	y	-0.07918	19	21	1	-16.92	IOL	y
183	EXG	69	M	0.5	192.5	0	1	y		0.69897	17	47	4	-20.17	IOL	y
184	EXG	77	M	0.55	273.5	0	2	y	n	0.045757	26	26	5	-29.8	y	n
185	EXG	79	F	0.42	325	0	2	y	n	0.69897	48	48	4	-13.12	IOL	y
189	POAG	62	M	0.5	355.5	0	2			-0.07918	17	25	3	-19.29	IOL	y
191	control	76	M	0.57	256.5	0	1	y	y	-0.07918	14	14	0		IOL	y
194	POAG	69	F	0.405	380.5	0	2	y	n	0.39794	22	22	3	-12.18	y	n
195	POAG	70	F	0.52	470.5	0	2	y	n	0.154902	21	21	3	-19.57	y	n
196	POAG	63	F	0.565	214	1	2	y	n	-0.07918	17	17	3	-19.23	y	n
199	EXG	79	M	0.52	606	0	2	n	y	0.154902	29	29	3	-18.88	IOL	y
200	POAG	47	F	0.425	594.5	0	3	y	n	-0.07918	19	22	2	-0.77	y	n
201	EXG	89	F	0.29	200.5	0	2	y	n	1.69897	19	54	0	-29.98	IOL	y
202	EXG	72	F	0.455	603	0	3	n	n	2.60206	47	47	4	-29.47	IOL	y
203	POAG	87	M	0.49	340	0	3			0.045757	5	24	3	-27.64	IOL	y
206	control	78	F	0.505	399.5	0	3	y	y	0.30103	14	14	0		y	n
209	EXG	70	F	0.38	520	0	1	n	n	0.221849	21	21	4	-21.31	IOL	y
210	EXG	74	F	0.49	514.5	0	0	n	n	-0.07918	37	37	4	-20.22	IOL	y
211	POAG	86	M	0.43	405	0	2	n	y	0.522879	14	14	4	-11.82	y	n
212	EXG	62	F	0.44	347	0	0	n	n	0	19	19	3	-7.3	y	n
214	POAG	82	F	0.465	375.5	0	2	y	n	0.09691	30	30	4	-6.63	IOL	y
215	EXG	67	F	0.455	380	0	3	y	n	0.045757	21	21	3	-5.35	y	n
216	POAG	86	F	0.475	383.5	0	3	y	y	0.045757	35	35	3	-29.15	IOL	y
217	EXG	77	F	0.545	331.5	0	3			0.221849	13	39	2	-21.34	IOL	y
218	control	74	M	0.5	413	0	2	n	y	0.39794	12	12	0		y	n
219	control	80	M	0.48	258.5	0	2	y	n	0.045757	14	14	0		y	n
221	EXG	61	F	0.525	475.5	0	2	n	n	0.69897	21	21	2	-8.71	IOL	y
222	POAG	76	M	0.42	444	0	2			0.221849	10	25	3	-28.85	IOL	y
223	POAG	66	M	0.75	238	0	2	y	n	2.69897	4	16	0	-17.97	y	y
224	EXG	83	F	0.62	345.5	0	2	y	n	1.221849	31	31	5	-29.19	y	n
226	POAG	64	F	0.38	355.5	0	2	n	n	-0.07918	17	19	3	-3.46	y	n
228	POAG	69	M	0.585	218.5	0	0	y	y	1	12	42	2	-29.56	IOL	y
230	POAG	71	M	0.355	328.5	1	2	y	y	-0.07918	13	19	3	-6.49	IOL	y
231	POAG	55	M	0.38	203	0	2			0	11.7	50	3	-29.28	IOL	y
234	POAG	53	F	0.6	313.5	0	1			-0.07918	14	23	2	-2.1	IOL	y
236	POAG	66	F	0.44	469.5	0	2	y	n	0	20	20	3	-11.27	y	n
237	POAG	69	F	0.375	514	0	2	y	n	0.30103	15	31	3	-21.9	y	n
241	EXG	66	M	0.315	213	0	2	y	n	0.221849	22	22	3	-21.3	y	n
242	EXG	71	M	0.505	304.5	0	2	y	y	0.045757	14	14	2	-18.71	IOL	y
244	POAG	64	F	0.4	794	0	2	y	y	-0.07918	17	17	2	-10.19	IOL	y
245	POAG	72	M	0.385	338.5	0	2	y		0.045757	11	24	2	-27.22	IOL	y
246	EXG	73	M	0.605	334.5	0	0			-0.07918	15	17	3	-2.27	IOL	y
247	EXG	79	F	0.47	431	0	2	y	y	0.154902	22	22	3	-8.25	IOL	y
248	POAG	66	F	0.86	240.5	0	2			1.09691	7	17	0	-20.99	IOL	y
249	control	78	M	0.38	303	1	3	n	y	0.09691	16	16	0		y	n
250	POAG	54	F	0.365	226	0	2	y	n	-0.07918	18	18	0	-0.72	y	n
252	control	61	M	0.435	595.5	0	2			-0.07918			0		y	n
254	POAG	79	F	0.5	378.5	0	1	y	y	1.045757	15	17	3	-25.77	IOL	y
255	POAG	61	M	0.46	252	1	0			0.69897	11	21	4	-9.93	IOL	y
256	EXG	85	M	0.675	148.5	1	0	y	n	0	13	26	2	-16.04	IOL	y
257	POAG	61	M	0.525	363	0	0	n	y	1.39794	14	36	4	-26.36	IOL	y
258	POAG	79	F	0.485	527.5	0	2			-0.07918	10	15	3	-21.03	y	n
259	POAG	67	F	0.385	412	0	2	y	n	0	9	16	1	-24.78	IOL	y
261	EXG	66	M	0.49	255.5	1	2	y	n	-0.07918	11	19	3	-12.02	IOL	y
264	POAG	42	F	0.325	265.5	1	2	n	n	-0.07918	17	22	4	-8.24	y	n
265	POAG	65	M	0.485	362	0	3	y	n	0.154902	13	15	3	-13.88	y	n
268	POAG	79	M	0.455	395.5	0	2	y	y	0.09691	15	15	3	-12.1	y	n
269	POAG	69	F	0.315	334.5	0	1	n	n	0	19	19	0	-4.67	y	n
271	POAG	60	M	0.495	249	0	2			0.30103	28	28	2	-16.26	y	n
272	EXG	86	M	0.465	166.5	0	1			1.39794	11	25	3	-23.87	IOL	y
273	POAG	66	M	0.375	206.5	0		y	n	-0.07918	15	17	3	-20.76	IOL	y
274	POAG	67	M	0.385	175	0	2	n	n	-0.07918	31	31	1	-12.75	IOL	y
275	POAG	53	M	0.42	122	0	2	y	n	-0.07918	21	21	1	-17.12	IOL	y
278	POAG	50	M	0.495	428.5	0	3	n	n	-0.07918	19	23	4	-9.59	IOL	y
279	EXG	68	F	0.5	248	1	2			-0.07918	13	28	3	-2.49	y	n
280	POAG	66	F	0.455	338.5	0	2	y	n	1	12	20	1	-17.71	IOL	y
281	POAG	80	M	0.505	383	0	3	n	y	-0.07918	15.7	29	3	-11.45	IOL	y
282	POAG	73	M	0.49	151.5	0	2		y	-0.07918	15	21	4	-15.85	IOL	y
284	POAG	69	M	0.505	123.5	0	1	y	y	-0.07918	19	22	3	-22.59	IOL	y
287	control	82	F	0.395	463.5	0	3	n	n	0			0		IOL	y
290	POAG	77	F	0.325	537	0	2	y	n	0.221849	20	20	2	-19.54	IOL	y
291	POAG	57	F	0.495	265.5	0	2	y	n	0.30103	15	15	3	-13.54	y	n
292	EXG	87	F	0.47	291	0	2	y	y	2.60206	23	23	1	-30.29	IOL	y
293	POAG	71	M	0.5	243	0	3	y	n	0.39794	42	44	3	-15.48	IOL	y
294	EXG	79	M	0.53	202	0	3	n	n	0.522879	27	27	3	-21.58	IOL	y
295	POAG	64	M	0.455	394.5	0	2	n	n	0.30103	19	27	4	-30.23	y	y
296	EXG	76	F	0.445	305	0	1	n	y	0	15	29	2	-5.42	IOL	y
297	EXG	82	F	0.52	373.5	0	3	y	n	0.39794	21	21	3	-7.31	IOL	y
299	EXG	78	F	0.74	317	0	3	n	n	0	13	17	1	-2.05	IOL	y
300	control	72	F	0.555	409.5	0	0	n	n	0.154902	12	12	0		y	n
301	POAG	74	M	0.47	427	0	2	y	n	-0.07918	17	31	2	-6	IOL	y
302	POAG	79	F	0.37	340.5	0	2			1.39794	20	30	2	-27.86	IOL	y
303	POAG	73	F	0.375	572.5	0	0			-0.07918	15	18	2	-13.47	IOL	y
304	POAG	62	F	0.46	182.5	0	0			0.522879	17	25	0	-20.18	IOL	y
305	POAG	58	F	0.355	467	0	3	n	n	-0.07918	20	20	3	-6.51	IOL	y
306	POAG	62	F	0.48	309.5	0	3	n	n	0	12	17	3	-22.59	IOL	y
308	EXG	78	M	0.665	140.5	0	0	y	y	-0.07918	12	26	3	-28.4	IOL	y
309	POAG	73	M	0.34	269	0	2			-0.07918	11	20	3	-22.26	IOL	y
310	POAG	35	M	0.42	170	1	0	n	n	0.154902	22	57	4	-25.47	y	y
311	POAG	69	F	0.39	239	0	2	y		0.522879	11	23	0	-7.62	y	y
312	POAG	77	M	0.45	325	0	2	y		-0.07918	24	24	2	-15.28	IOL	y

313	POAG	59	M	0.38	306	0	2	n	n	0	10	15	4	-10.31	y	n
314	POAG	61	F	0.355	405.5	0	2			-0.07918	17	19	3	-4.03	IOL	y
315	POAG	64	M	0.435	525	0		y	n	-0.07918	17	17	3	-8.92	y	n
316	POAG	64	M	0.505	410.5	0	3	y	n	-0.07918	16	16	4	-10.99	y	n
317	POAG	68	M	0.36	438.5	0	3	y	n	0.39794	17	19	1	-10.04	y	n
320	POAG	64	F	0.53	460	0	2	n	n	0.221849	23	23	1	-23.27	y	n
323	POAG	64	F	0.48	402	0	3	y	n	0.154902	20	20	4	-13.76	y	n
324	EXG	86	M	0.51	385.5	0	3	n	n	0.823909	21	21	4	-11.06	y	n
326	POAG	51	F	0.435	323	0	2			0.522879	16	18	3	-24.65	IOL	y
327	control	67	F	0.3	480	0	2	n	n	0.09691	18	18	0		y	n
330	POAG	51	M	0.42	253.5	0	0			-0.07918	19	58	4	-1.51	y	y
332	EXG	59	F	0.355	343.5	0	2	y		0.30103	9	17	3	-27.75	IOL	y
333	EXG	75	F	0.645	369.5	0	0			0	11	11	3	-5.77	IOL	y
334	POAG	87	M	0.4	225.5	0	1	n	n	0.522879	13	13	0	-29.61	IOL	y
340	EXG	68	F	0.3	573.5	0	3	y	n	-0.07918	13	39	1	-13.51	IOL	y
341	EXG	76	M	0.43	532.5	0	3	y	n	0.221849	17	28	0	-24.87	IOL	y
342	POAG	84	M	0.435	354.5	0	3	n	n	0.09691	13	14	3	-31.45	IOL	y
344	POAG	55	M	0.415	403			n	n	0	12	12	2	-21.48	y	n
345	EXG	87	F	0.505	268	0	3	y	y	0.39794	29	30	4	-30.29	IOL	y
346	POAG	65	M	0.495	368	0	3	y	n	-0.07918	25	25	3	-9.63	y	n
348	POAG	73	M	0.45	246	0	2			0.522879	22	31	3	-20.11	IOL	y
350	control	80	M	0.455	417	0	3	y	y	0.221849	10	10	0		y	n
355	POAG	59	M	0.455	216	0	2	n	n	0.30103	13	23	3	-9.79	IOL	y
356	EXG	81	M	0.42	201	0	2			0	4	26	0	-29.17	IOL	y
360	POAG	44	F	0.37	347	0	1			0	13	13	3	-13.03	y	n
361	POAG	70	F	0.255	434	0	3	y		1	15	25	2	-26.44	IOL	y
362	POAG	69	F	0.41	265.5	0	2	y		1.221849	11	38	3	-28.7	IOL	y
363	POAG	63	F	0.4	432	0	3	y	n	0.39794	19	26	3	-9.59	IOL	y
365	POAG	55	M	0.365	136	1	0	y	n	-0.07918	10	22	1	-10.32	y	y
367	EXG	83	M	0.355	197.5	0	0			0.30103	16	19	1	-27.44	IOL	y
369	control	84	F	0.475	259.5	0	2	y		0.30103	12	12	0		y	n
373	POAG	81	F	0.47	208.5	0	2			0.045757	12	12	1	-30.67	IOL	y
376	EXG	88	M	0.505	450	0	0	y	n	1.522879	43	43	3	-26.82	IOL	y
377	POAG	60	M	0.4	493.5	1	2	n	n	0	13	14	3	-18.85	y	n
379	EXG	76	F	0.54	240.5	1	2	y	n	0.221849	15	15	3	-13.67	y	n
382	EXG	71	F	0.555	469.5	0	2	n	n	0.39794	15	16	3	-15.34	IOL	y
384	EXG	73	F	0.47	402.5	0	2	y	n	0.221849	23	23	3	-23.83	IOL	y
385	EXG	73	M	0.475	533	0	2			0	22	29	3	-11.86	IOL	y
387	POAG	66	M	0.435	191	0	0	n	n	0.522879	15	27	0	-31.59	y	y
389	EXG	81	F	0.325	241.5	0	3			-0.07918	9	45	2	-13.35	IOL	y
391	EXG	79	M	0.3	530.5	0	2	y	n	2.60206	18	36	3	-33.52	IOL	y
393	POAG	79	F	0.405	329	0	2	y	n	0	15	19	2	-27.48	IOL	y
394	EXG	69	M	0.965	295.5	0	2	y	n	-0.07918	11	21	2	-14.57	IOL	y
395	EXG	88	M	0.6	271	0	1	y	n	0.39794	11	24	2	-25.99	IOL	y
400	POAG	80	M	0.505	162.5	0	1	y	n	0.09691	10	11	3	-26.47	IOL	y
403	POAG	52	F	0.405	553	0	2			0.823909	11	20	4	-12.22	y	n
404	POAG	86	M	0.36	157.5	0	2	y	n	0.221849	14	14	1	-18.97	y	n
405	POAG	80	F	0.53	382.5	0	1			-0.07918	12	15	3	-13.33	IOL	y
406	POAG	78	M	0.52	463	0	1	n	n	0.221849	15	15	1	-2.32	y	n
408	control	70	F	0.595	230.5	0	1	n	n	-0.07918	14	14	0	-1.15	y	n
411	EXG	48	M	0.445	169.5	0	1	n	n	0.154902	7	29	2	-10.89	IOL	y
419	POAG	59	F	0.4	319.5	0	2	n	n	-0.07918	15	15	2	-10.09	IOL	y
420	POAG	71	M	0.445	368	0	2	y	y	0.154902	14	14	1	-5.07	IOL	y
422	EXG	86	M	0.58	350.5	0	3	y	y	0.30103	13	29	2	-17.2	IOL	y
424	POAG	58	M	0.575	302.5	0	3	y	n	0	47	47	4	-2.06	y	y
427	EXG	65	F	0.42	451	0	3	n	n	0.09691	16	58	4	-22.46	y	n
428	EXG	86	F	0.55	186	0	1	y	y	0.522879	11	34	1	-20.96	IOL	y
429	EXG	67	M	0.45	234	1	0	n	n	0.221849	15	19	2	-20.42	IOL	y
430	POAG	65	F	0.395	668	0	3	n	n	-0.07918	17	23	4	-11.39	IOL	y
431	POAG	68	F	0.585	247.5	0	2			-0.07918	11	26	2	-11.48	IOL	y
438	POAG	60	M	0.545	373	0	2			0.045757	13	49	1	-15.02	IOL	y
440	POAG	69	M	0.515	360.5	0	3	y	n	0.522879	11	17	2	-28.76	IOL	y
441	POAG	74	M	0.505	590	0	3			0.09691	5	27	0	-23.97	IOL	y
442	POAG	69	F	0.33	551.5	0	3	n	n	0.30103	11	17	1	-25.21	IOL	y
444	EXG	74	M	0.53	268.5	0	2	y	n	0.09691	25	25	3	-21.94	IOL	y
445	EXG	85	F	0.575	347	0	2	y	n	0.39794	19	19	1	-4.97	y	n
447	EXG	89	F	0.52	478.5	0	2	y	n	0.221849	26	26	4	-29.78	IOL	y
449	EXG	88	F	0.48	357	0	2	n	n	0.69897	21	23	0	-26.64	y	n
452	POAG	71	M	0.43	285	1	3	y	n	-0.07918	13	24	3	-9.08	IOL	y
457	POAG	76	M	0.53	218.5	0	0			-0.07918	9	38	0	-15.01	IOL	y
458	POAG	63	M	0.455	508.5	0	3			0.154902	14	18	0	-12.68	y	n
461	POAG	73	F	0.46	428	0	2		y	-0.07918	15	19	2	-9.24	IOL	y
463	POAG	61	F	0.5	186.5	0	2	n	n	0.30103	21	21	2	-6.03	y	y
464	EXG	70	M	0.47	290	0	2	y	n	0.09691	13	25	4	-26.02	IOL	y
465	POAG	75	M	0.595	290.5	0	2	n	n	0	19	32	2	-0.51	y	n
466	POAG	69	M	0.48	302.5	0	2	n	n	0	15	15	4	-15.52	y	n
468	POAG	45	M	0.465	220.5	0	2	n	n	-0.07918	13	13	1	-11.31	y	n
469	POAG	61	M	0.525	363	0	1	n	y	1.39794	14	31	4	-26.36	IOL	y
470	POAG	61	M	0.485	258.5	0	2	y	y	-0.07918	11	19	3	-18.97	IOL	y
471	EXG	91	M	0.545	344.5	0	2	n	n	0.154902	15	31	3	-29.93	IOL	y
473	EXG	83	F	0.53	272.5	0	0	y	n	2	15	15	4	-29.18	IOL	y
474	EXG	75	F	0.46	298	0	2	y	n	-0.07918	11	24	2	-26.66	IOL	y
476	POAG	59	F	0.45	226.5	0	0	y	y	0.09691	25	25	4	-18.38	y	n
478	POAG	72	F	0.41	268.5	0	1	n	n	0.09691	13	15	2	-14.28	IOL	y
479	POAG	70	M	0.42	267	0	2			0.69897	11	25	3	-28.19	IOL	y
480	EXG	70	M	0.575	115.5	0	1	y	y	1.045757	4	29	0	-23.41	IOL	y
481	POAG	59	M	0.45	286	0	2	n	n	0.30103	14	27	3	-27.27	y	y
482	EXG	71	F	0.355	366	0	2	n	n	0.39794	17	49	2	-26.16	IOL	y
483	POAG	67	F	0.44	369	0	3	n	n	-0.07918	23	23	3	-2.22	y	n
484	EXG	83	M	0.435	334.5	0	2	y	n	0.39794	18	18	4	-4.51	IOL	y
485	POAG	72	M	0.495	342	0	3	n	y	0.30103	23	23	4	-22.76	IOL	y
487	POAG	71	F	0.26	524	0	2	y	n	-0.07918	27	27	4	-9.12	y	n
488	POAG	80	F	0.51	460.5	0	2	n	y	0.39794	11	11	3	-18.22	y	n
490	POAG	73	M	0.55	181.5	0	2	y	y	0.045757	20	20	3	-3.51	IOL	y
491	POAG	66	F	0.51	263	0	2	n	n	0.69897	11	19	2	-29.57	IOL	y
492	POAG	71	M	0.63	268	0	1	y	n	-0.07918	15	26	3	-11.34	IOL	y
495	EXG	65	F	0.535	319.5	0	2	y	y	-0.07918	12	31	2	-15.77	y	n

498	EXG	73	F	0.47	333.5	0	1	y	y	0.09691	19	37	2	-19.7	IOL	y
502	POAG	68	M	0.495	647.5	0	2			-0.07918	11	17	3	-18.04	y	n
503	POAG	60	F	0.455	255	1	2	y		-0.07918	13	16	4	-15.6	y	n
505	POAG	68	F	0.415	395.5	0	2			0	11	18	3	-9.25	y	n
506	EXG	67	M	0.44	613.5	0	2	n	n	0	23	23	4	-3.18	y	n
507	POAG	68	F	0.415	369.5	0	2			0.154902	11	13	4	-19.32	y	n
508	POAG	65	M	0.375	409.5	0	1	n	y	0	14	14	3	-16.73	y	n
511	EXG	74	F	0.525	648.5	0	3	y	n	0	16	33	3	-10.48	IOL	y
512	POAG	78	F	0.455	254.5	0	3	y	n	0.154902	12	12	3	-25.3	IOL	y
513	POAG	62	F	0.37	551.5	0	3			-0.07918	15	17	3	-22.81	IOL	y
515	POAG	52	M	0.385	348	0	3	n	n	0.39794	17	17	3	-19.5	IOL	y
516	EXG	69	M	0.41	313.5	0	3	n	n	0.154902	17	17	4	-11.01	y	n
517	EXG	90	F	0.505	413.5	0		y	n	1	9	58	4	-29.48	IOL	y
519	POAG	59	M	0.43	355	1	2	y	n	0.09691	14	25	3	-19.23	IOL	y
520	EXG	75	F	0.56	391	0	2			-0.07918	12	23	2	-0.96	y	n
521	POAG	65	F	0.41	266	0	1	n	n	-0.07918	11	11	4	-10.64	IOL	y
522	EXG	63	M	0.44	229.5	0	1			-0.07918	13	28	3	-3.32	IOL	y
523	EXG	69	M	0.385	215	0	2			0.221849	12	38	0	-3.71	IOL	y
524	control	77	F	0.465	372.5	0	2	n	n	0.30103			0		y	n
529	EXG	67	M	0.46	285	1	2			0	19	21	0	-2.14	y	n
530	POAG	48	F	0.425	449.5	0	2			-0.07918	11	22	4	-17.97	y	n
531	EXG	64	M	0.375	291.5	0	2	n	n	0.09691	16	37	3	-28.26	IOL	y
532	POAG	77	M	0.405	447.5	0	2	n	n	0.09691	14	20	3	-24.92	IOL	y
533	POAG	64	M	0.46	242.5	0	2	n	n	-0.07918	17	21	3	-20.14	IOL	y
534	POAG	65	M	0.38	220	1	2	y		-0.07918	14	21	1	-16.89	IOL	y
536	POAG	72	M	0.5	192.5	1	2	y		0.522879	9	30	3	-28.52	IOL	y
537	control	71	M	0.435	130.5	1	2	n	n	0.09691			0		y	n
538	EXG	73	M	0.455	261	0	3			-0.07918	25	27	1	1.25	IOL	y
540	EXG	71	M	0.205	538	1	2	y	n	-0.07918	23	23	5	-13.76	y	n
541	POAG	58	F	0.405	320	0	2	y	y	-0.07918	14	16	3	-26	y	n
544	POAG	68	M	0.44	347	0	2			0.69897	11	16	3	-26.8	y	y
547	EXG	72	M	0.43	586.5	0	2	n	n	-0.17609	9	32	3	-14.26	IOL	y
548	POAG	70	M	0.495	186	0	2			-0.07918	17	19	4	-9.39	y	n
549	POAG	63	M	0.32	146.5	1	1	y		0.045757	13	19	4	-20.73	IOL	y
550	EXG	68	M	0.545	196	1	3	y		-0.07918	19	29	0	-1.02	y	y
551	POAG	73	F	0.44	256	0	3	y	n	0	14	21	4	-13.34	IOL	y
554	EXG	69	M	0.55	386.5	0	1	n	n	-0.07918	13	46	1	-30.23	IOL	y
555	EXG	84	F	0.4	593.6667	0	2			0.39794	8	42	3	-29.21	IOL	y
556	POAG	75	M	0.455	495.5	1	0	y	n	0.522879	15	21	4	-19.14	IOL	y
557	EXG	67	M	0.41	327	1	2		y	-0.17609	11	51	0	1.11	IOL	y
560	POAG	58	F	0.445	236.5	0	2			0	11	17	1	0.13	IOL	y
561	EXG	81	F	0.395	316.5	0	2			0.39794	17	14	2	-28.9	IOL	y
562	POAG	77	M	0.4	364.5	0	2			0	17	21	3	-15.34	IOL	y
563	POAG	59	M	0.47	270	0	3	y	n	-0.07918	11	20	3	-14.8	y	y
565	POAG	65	F	0.565	356.5	0	2			0.221849	8	8	0	-26.87	IOL	y
572	POAG	81	F	0.52	401	0	3	y	n	0	15	20	1	-15.81	IOL	y
573	POAG	62	M	0.37	378.5	0	3			-0.07918	11	16	4	-18.52	y	n
576	control	82	F	0.49	418.5	0	2	y	y	0.69897			0		y	n
577	POAG	70	M	0.46	234	0	0			-0.07918	11	23	3	-10.1	y	n
583	POAG	79	M	0.52	257	0	2		y	0	13	22	2	-10.01	IOL	y
584	POAG	60	M	0.345	530	0	2			-0.07918	19	22	4	-24.21	IOL	y
586	POAG	72	F	0.515	318.5	0	3	y		0.154902	17	17	4	-25.1	IOL	y
588	POAG	76	M	0.485	241	0	2	y		0.221849	10	16	3	-18.94	IOL	y
589	POAG	66	F	0.535	417.5	0	2		y	0.522879	13	34	4	-20.02	IOL	y
590	POAG	67	M	0.4	204	0	1			0.09691	11	21	3	-13.51	IOL	y
592	POAG	70	M	0.34	163	1	1			0.30103	15	25	3	-15.1	y	n
593	POAG	74	F	0.45	457	0	3			0.154902	6	25	0	-28.6	IOL	y
595	POAG	62	M	0.395	300.5	0	1	y	n	1	15	15	4	-33.89	IOL	y
596	POAG	41	F	0.32	290	0	3			-0.07918	17	45	0	-3.87	y	n
597	POAG	61	M	0.435	210	1	0			0.39794	13	30	4	-31.14	IOL	y
599	POAG	84	F	0.455	347	0	3	y	y	0.045757	17	25	2	-8.66	IOL	y
600	POAG	66	M	0.475	317.5	0	1	y	n	-0.07918	15	24	3	-6.79	IOL	y
602	POAG	71	M	0.495	236.5	1	1			-0.07918	11	18	2	-19.97	y	n
603	POAG	77	M	0.43	573	0	3			0	16	29	4	-18.5	IOL	y
606	EXG	82	F	0.475	290.5	0	2			0.39794	9	44	0	-28.74	IOL	y
608	POAG	61	M	0.45	378.5	0	2	n	n	-0.07918	9	17	4	-23.85	IOL	y
609	POAG	48	F	0.32	206	1	0			0	15	21	3	-6.08	y	y
611	POAG	67	M	0.365	257	0	0	n	n	0.09691	14	19	3	-19.73	IOL	y
612	POAG	71	M	0.345	349.5	0	2		y	0	13	34	4	-7.07	IOL	y
613	POAG	63	F	0.44	363.5	0	3			1.09691	10	51	0	-18.18	y	y
619	POAG	77	F	0.4	115.5	0	2	y	n	0.09691	13	13	2	-6.65	IOL	y
620	EXG	83	M	0.525	316.5	0	2	n	n	0.30103	14	14	5	-17.32	y	n
621	EXG	82	M	0.53	168	0	1	y	n	0.30103	21	21	4	-29.91	IOL	y
622	POAG	77	F	0.395	283	0	2			0	12	12	2	-7.1	y	n
624	POAG	52	M	0.415	292	0	2			0	14	15	4	-24.1	y	n
625	POAG	58	M	0.64	181.5	0	2	n	n	-0.07918	14	18	4	-12.76	y	y
628	EXG	69	M	0.28	321.5	1	3		y	0.154902	15	41	3	-28.04	IOL	y
629	POAG	61	F	0.48	502.5	0	2			-0.07918	11	45	0	-8.86	y	y
630	POAG	59	M	0.455	347.5	0	3			-0.07918	14	47	0	-19.76	y	n
631	POAG	50	M	0.495	428.5	0	3	n	n	-0.07918	19	23	4	-9.59	y	y
632	POAG	84	M	0.455	132	0	3	y	n	0.221849	11	15	3	-23.57	IOL	y
633	POAG	55	F	0.415	340	0	3	y	n	0.09691	13	19	1	-18.25	y	y
634	POAG	78	F	0.355	671	0	3	y	n	-0.07918	12	17	3	-10.16	IOL	y
639	control	83	F	0.525	446	0	1			0.30103	18	18	0		y	n
643	POAG	78	M	0.375	575	0	2	n	y	0	10	24	2	-13.34	IOL	y
645	POAG	57	M	0.52	230.5	1	2	n	n	-0.07918	19	19	4	-22.49	y	n
646	POAG	81	M	0.455	481	0	3			0.09691	12	18	0	-9.73	IOL	y
650	POAG	52	M	0.385	302.5	0	2			-0.07918	9	15	3	-13.82	y	n
652	POAG	88	M	0.39	238	0	2	y	n	0.154902	9	19	2	-20.27	IOL	y
655	POAG	70	M	0.335	511.5	0	2			0.221849	11	18	3	-7.72	IOL	y
664	POAG	76	M	0.4	284.5	0	2		y	-0.07918	17	19	1	-16.47	IOL	y
665	EXG	85	F	0.545	337.5	0	3	y	n	0	9	24	2	-7.08	IOL	y
667	EXG	85	M	0.48	85	0	1	y	n	0.154902	19	19	3	-19.2	y	n
668	POAG	57	M	0.57	230	0	2			1.39794	12	23	4	-31.01	y	y
669	POAG	67	M	0.29	273	1	1	n	n	0.09691	15	15	2	-5.42	IOL	y
672	EXG	85	M	0.53	220.5	0	2	y	n	2	35	35	2	-30.47	IOL	y
673	EXG	80	F	0.39	554	0	2	n	n	-0.20412	21	21	1	-12.24	IOL	y

674	POAG	65	F	0.515	355.5	0	3	y		0	15	40	0	-15.24	IOL	y
676	control	70	F	0.395	224.5	0	3	y	n	0.045757			0		y	n
681	EXG	92	F	0.545	183.5	0	2			0.30103	11	27	4	-28.64	IOL	y
682	EXG	64	M	0.49	281.5	0	1	n	n	0.522879	14	25	2	-22.35	IOL	y
683	control	78	F	0.4	258.5	0	2	y	n	1	13	13	0		y	n
684	EXG	82	F	0.505	410.5	0	2			0.522879	8	30	3	-15.41	IOL	y
687	POAG	50	F	0.33	392	1	3			-0.07918	17	20	3	-12.14	y	n
688	POAG	56	M	0.4	202	0	2	n	y	-0.07918	11	15	2	-2.55	y	n
690	POAG	69	M	0.31	147	1	2			0	8	21	4	-29.84	IOL	y
693	POAG	81	F	0.45	497	0	3	y	n	0.045757	12	16	2	-10.51	IOL	y
696	POAG	81	M	0.54	166.5	0	2		y	0	11	20	3	-15.01	IOL	y
697	POAG	66	M	0.475	139	0	1	y	n	-0.07918	20	20	4	-19.26	IOL	y
698	EXG	76	M	0.545	288.5	0	3	n	n	0	10	27	3	-5.23	IOL	y
702	POAG	73	F	0.495	372.5	0	3			0	15	19	2	-1.08	y	n
703	POAG	71	F	0.325	334.5	0	2			0.045757	14	19	1	-10.62	IOL	y
704	EXG	87	M	0.54	301	0	2			0.221849	27	32	3	-29.52	IOL	y
705	POAG	66	M	0.405	445.5	0	2			0	10	12	3	-7.66	y	n
711	POAG	55	F	0.45	635	0	3			-0.07918	13	20	2	-0.94	y	n
713	EXG	77	F	0.5	331.5	0	2	y		-0.07918	7	55	2	-12.58	IOL	y
718	POAG	70	M	0.445	213	0	1	y		-0.07918	13	22	3	-24.43	IOL	y
719	POAG	71	M	0.45	484	0	3			-0.07918	15	21	1	-3	y	n
723	control	77	F	0.535	222.5	0	1			0.39794	11	11	0		y	n
724	control	79	M	0.485	400	0	2			0.09691	13	13	0		y	n
729	control	80	M	0.43	252.5	0	1	y	n	0.154902	14	14	0		y	n
736	POAG	33	F	0.475	408	0	0	n	n	-0.07918	17	17	0	-3.15	y	n
738	POAG	61	M	0.455	465.5	0	2	y	n	0.045757	13	13	4	-10.16	y	n
739	POAG	72	M	0.425	312.5	0	2	y		0.221849	5	16	0	-27.38	IOL	y
740	EXG	64	M	0.46	287	0	2	y	n	-0.07918	27	27	0	-2.37	y	n
741	POAG	51	F	0.5	262.5	0	2			-0.07918	12	18	3	-13.85	y	y
743	POAG	79	M	0.42	349	0	2	y		0.522879	19	19	4	-21.95	IOL	y
748	POAG	43	M	0.45	534	0	2	n	n	-0.07918	11	14	1	-3.8	y	n
749	control	78	F	0.485	434.5	0	3			0.045757	12	12	0		y	n
750	control	71	F	0.485	297.5	0	2			0.09691	14	14	0		y	n
751	control	78	F	0.36	445	0	3	y	y	0.045757	14	14	0		y	n
756	control	77	M	0.515	434	0	2	y	y	0.09691	16	16	0		y	n
757	control	88	F	0.495	349.5	0	2	y	n	0.39794	16	16	0		y	n
758	EXG	69	F	0.63	330	0	2	n	n	-0.07918	15	20	3	-12.92	IOL	y
759	EXG	72	M	0.44	235.5	0	2			-0.07918	15	28	0	-12.82	IOL	y
760	POAG	51	F	0.49	287	0	2			-0.07918	11	22	3	-12.51	y	y
762	POAG	61	F	0.505	344.5	1	2			-0.07918	12	17	3	-16.6	y	n
766	POAG	72	M	0.53	235	0	1	y	n	-0.07918	14	17	3	-9.51	IOL	y
769	control	81	F	0.335	308.5	0	2	n	n	0.30103			0		IOL	y
772	EXG	76	M	0.36	291.5	0	1		y	0.09691	10	59	3	-25.64	IOL	y
773	control	84	M	0.53	170	0	1	n	n	0.154902			0		y	n
775	POAG	67	M	0.345	157.5	1	2	y	y	0.045757	8	10	4	-26.68	IOL	y
776	POAG	69	M	0.32	370.5	0	2			0.221849	11	15	2	-4.47	y	n
778	EXG	49	M	0.375	258.5	1	0	n	n	-0.07918	15	15	1	-3.18	y	n
779	EXG	71	M	0.41	482.5	0	2	y	y	0.823909	9	30	3	-30.58	IOL	y
781	POAG	59	M	0.47	327.5	0	3			0.09691	12	21	3	-19.84	IOL	y
783	POAG	73	F	0.405	447.5	0	3		y	0.154902	11	17	1	-23.95	IOL	y
784	control	53	F	0.3	428	0	2		y	0.09691	18	18	0		y	n
785	EXG	83	F	0.565	274.5	0	2	y	n	0.221849	11	20	3	-30.43	IOL	y
787	POAG	71	M	0.395	318	0	2	n	n	0.823909	10	10	1	-8.45	y	n
788	control	81	M	0.595	364.5	0	3			0.221849			0		y	n
791	POAG	69	M	0.35	188	1	2	y	n	0.045757	5	13	0	-16.65	y	y
795	POAG	66	F	0.43	287.5	0	2	n	n	-0.07918	16	22	3	-8.5	IOL	y
796	EXG	81	F	0.415	448	0	2	y	n	-0.07918	11	20	3	-19.28	IOL	y
798	control	64	M	0.52	161.5	0	1			-0.07918	12	12	0		y	n
799	control	70	M	0.5	309	0	2	y	n	0.30103	20	20	0		y	n
800	control	69	M	0.42	239.5	1	2			0.154902	18	18	0		y	n
802	POAG	82	F	0.345	116.5	0	1			0.09691	11	17	3	-29.3	IOL	y
808	control	83	M	0.6	220	0	1	y	y	0.39794			0		y	n
810	control	70	F	0.41	237.5	0	2	n	n	0.154902	13	13	0	-1.64	y	n
812	control	69	M	0.42	320.5	0	2	n	n	0.154902			0		y	n
815	POAG	57	M	0.385	249.5	0	1	n	n	-0.07918	7	9	0	-13.39	y	n
816	POAG	74	M	0.445	278	0	3			0.522879	11	18	5	-30.22	IOL	y
818	POAG	80	F	0.385	449	0	3	y	y	0.39794	13	24	2	-24.87	IOL	y
819	POAG	51	M	0.37	194.5	1	3	y	n	-0.07918	11	18	5	-13.64	y	n
823	control	70	M	0.33	164	0				0.69897			0		y	n
824	POAG	49	F	0.38	378.5	0	2			-0.07918	11	17	4	-21.29	y	n
826	EXG	78	M	0.58	306	0	2	y	n	-0.07918	11	17	3	-0.45	IOL	y
827	POAG	74	F	0.475	453.5	0	2	n	n	1	17	18	3	-13.26	IOL	y
829	control	76	M	0.38	376	0	0			0.09691		23	0		y	n
831	POAG	82	F	0.54	315	0	1	y	n	-0.07918	14	17	2	-12.01	IOL	y
833	POAG	83	F	0.42	422	0	2	y	y	0	11	22	2	-28.94	IOL	y
839	POAG	77	M	0.74	452.5	0	2			0.09691	13	17	4	-22.3	IOL	y
845	POAG	82	F	0.315	240	0	3	y	y	0.045757	13	27	2	-25.56	IOL	y
847	POAG	79	F	0.41	541	0	2	y	n	0	16	22	4	-15.9	y	n
849	POAG	67	M	0.42	223.5	1	2	y	n	0.221849	15	15	2	-15.06	y	n
850	POAG	49	F	0.38	275	0	2	n	n	-0.07918	20	20	0	-1.64	y	n
852	POAG	73	M	0.44	774	0	3	y		0.045757	14	19	3	-10.29	y	y
853	POAG	52	F	0.285	281	1	2	n	n	-0.07918	21	21	4	-8.77	y	n
854	POAG	69	F	0.44	415	0	3	n	n	0.30103	20	20	4	-9.73	y	n
855	EXG	77	M	0.49	417	0	3	y	n	1.69897	14	14	4	-26.98	y	n
858	control	51	M	0.495	264.5	0	2	n	n	-0.07918	20	20	0		y	n
859	control	74	F	0.325	502.5	0	3			-0.07918			0		y	n
861	control	73	F	0.3	139.5	0	1	y	n	0.045757			0		y	n
863	control	61	F	0.365	233.5	0	2	n	n	0.522879	12	12	0		y	n
864	POAG	47	M	0.435	206.5	1	2			-0.07918	13	23	3	-3.7	y	y
866	EXG	60	M	0.47	181.5	0	1	y		0.522879	9	51	2	-18.62	IOL	y
868	POAG	66	M	0.48	387	0	2	y	n	0.09691	16	39	2	-11.64	y	n
869	control	75	M	0.43	255.5	0	3	n	y	0.154902	14	14	0		y	n
872	POAG	69	M	0.37	959.5	0	2	y	y	1.30103	14	23	3	-29.86	y	n
873	POAG	70	F	0.405	329.5	0	3			0.154902	9	16	2	-26.07	IOL	y
874	EXG	86	M	0.395	199.5	1	2	n	n	0.69897	13	13	0	-5.68	y	n
875	EXG	72	F	0.455	398.5	0	3	n	n	-0.07918	39	39	4	-3.71	IOL	y
878	POAG	75	F	0.37	339.5	0	3			0.09691	19	19	3	-0.11	IOL	y

879	control	81	F	0.455	293	0	2		y	0.30103	12	12	0		y	n
881	EXG	82	M	0.645	407.5	0	2		y	1	6	28	0	-24.35	IOL	y
882	control	58	M	0.445	231.5	1	1		y	0.221849	18	18	0		y	n
883	control	69	M	0.27	182	1	1		y	0.154902	18	18	0		y	n
885	control	74	F	0.435	175.5	0	0	n	n	0.39794			0		y	n
891	POAG	57	F	0.38	300	0	2	y	n	-0.07918	12	23	3	0.79	y	y
892	POAG	58	M	0.43	378.5	0	2	y	y	-0.07918	8	21	0	-12.93	y	y
896	POAG	64	F	0.455	428	0	2	n	n	0.39794	9	19	0	-11.25	y	y
897	control	92	F	0.47	439	0	3			0.39794	10	10	0		IOL	y
899	POAG	71	M	0.48	324	0	2	y	n	1.69897	20	20	0	-7.85	y	n
900	POAG	58	M	0.425	388.5	0	2	y	n	0.30103	13	14	2	-9.22	y	n
902	POAG	54	M	0.435	367.5	0	2			0.045757	10	18	3	-14.33	y	n
903	POAG	51	M	0.36	662	0	3			-0.07918	12	19	4	-6.54	y	n
904	POAG	63	M	0.41	725	0	3	y	y	0	14	14	3	-10.77	y	n
906	POAG	65	M	0.375	544	0	2	n	n	-0.07918	11	11	3	-6.72	y	n
908	EXG	77	F	0.56	281.5	0	2		y	2.60206	16	16	4	-29.64	y	n
909	POAG	56	F	0.425	249	0	0	n	n	-0.07918	14	20	4	-5.52	y	n
914	POAG	56	M	0.425	295	0	1	n	n	0.522879	16	31	3	-24.21	y	y
915	POAG	53	F	0.385	377.5	0	2			-0.07918	14	16	2	-1.35	y	n
919	POAG	73	M	0.515	274.5	0	2	y		0	9	17	4	-18.61	y	y
923	control	71	F	0.365	315	0	2	n	n	0.154902	17	17	0		y	n
924	POAG	50	F	0.45	324	0	2	n	n	-0.07918	20	23	0	-4.43	y	n
925	POAG	76	M	0.52	250.5	0	1			-0.07918	7	12	3	-20	IOL	y
926	POAG	52	F	0.595	316.5	0	1	n	n	0.522879	11	11	0	-3.94	y	n
927	POAG	78	F	0.48	279.5	0	3			0.045757	12	19	2	-19.4	IOL	y
931	control	73	M	0.375	279.5	0	1			0.221849			0		y	n
938	EXG	86	F	0.64	233.5	0	1	y		0.154902	12	14	1	-17.5	y	n
941	control	90	M	0.515	305	0	2	n	y	0.522879			0		y	n
943	control	59	M	0.415	140.5	0		n	n	0.221849			0		y	n
946	POAG	68	M	0.53	278.5	0	2	y	n	0.39794	12	16	1	-7.61	y	n
947	control	80	F	0.5	198	0	3	y	n	0.154902	14	14	0		y	n
948	POAG	58	M	0.64	181.5	0	2	n	n	0	16	21	4	-12.76	y	y
952	POAG	55	M	0.445	254	1	0	n	n	-0.07918	25	25	0	-6.12	y	n
954	control	68	M	0.35	247	0	2	y	n	0.154902	14	14	0		y	n
955	control	92	M	0.315	389.5	0	2			1.221849	16	16	0		y	n
962	POAG	65	M	0.62	241	0	2	n	y	0.522879	11	11	3	-28.31	y	n
964	control	80	M	0.49	401.5	0	2	y	n	0.221849			0		y	n
965	control	70	F	0.43	316.5	0	1	y		0.221849			0		y	n
967	control	75	M	0.53	333	0	0	y	n	0.221849	14	14	0		y	n
968	control	68	M	0.38	339.5	1	1	y	y	0.221849	11	11	0		y	n
969	control	85	F	0.475	153.5	0	2	y	n	0.39794			0		y	n
971	POAG	76	M	0.34	373.5	0	1	n	n	0.154902	11	24	3	-19.17	IOL	y
972	POAG	56	F	0.375	513	0	2			-0.07918	13	18	4	-10.35	IOL	y
977	control	74	F	0.455	371.5	0	3	n	n	0.221849			0		y	n
983	POAG	50	M	0.465	279.5	0	2	n	n	-0.07918	15	15	1	-10.11	y	n
991	control	70	M	0.43	252.5	0	2	n	n	0.221849	10	10	0		y	n
997	control	82	M	0.425	246.5	0	1	n	n	0.154902			0		y	n
998	POAG	65	F	0.275	285	1	0	n	n	-0.07918		16	1	-5.89	y	n
1000	POAG	43	F	0.43	501.5	0	2			-0.07918	15	23	4	-13.29	y	y
1003	EXG	83	F	0.51	269	0	2	n	n	0	12	12	5	-16.59	IOL	y
1004	control	81	M	0.49	269	0	2	n	n	0.09691	17	17	0	-7.19	y	n
1007	POAG	84	F	0.435	299	0	3	y	n	1	12	31	4	-27.74	IOL	y
1010	EXG	85	M	0.48	427.5	0	2	y	n	0.69897	10	10	4	-32.97	IOL	y
1011	EXG	65	F	0.55	203.5	0	2	y	n	-0.07918	16	33	3	-24.12	IOL	y
1017	control	67	F	0.475	293.5	0	3	n	n	0.30103			0		y	n
1027	control	78	M	0.425	190.5	1	2	n	n	0.30103	12	12	0		y	n
1028	POAG	45	M	0.39	332.5	0	2	n	n	-0.07918	11	11	2	-10.06	y	n
1030	EXG	66	M	0.4	236.5	1	1	y	y	-0.07918	15	15	3	-5.08	IOL	y
1033	POAG	56	F	0.475	209.5	0	1			-0.07918	9	17	0	-12.7	y	n
1034	POAG	78	F	0.39	429.5	0	3	y	n	0.221849	9	9	2	-6.08	y	n
1035	control	67	M	0.345	242	0	1			0			0		y	n
1038	control	80	F	0.445	364	0	2			0.221849			0		y	n
1039	control	85	M	0.515	139.5	0	2		n	0.30103			0		y	n
1040	control	60	F	0.475	368.5	0	2	n	n	-0.07918			0		y	n
1041	control	64	F	0.39	377.5	0	2			-0.07918	14	14	0		y	n
1043	EXG	86	M	0.55	237.5	0	1			2.60206	26	40	4	-29.5	IOL	y
1052	control	82	M	0.46	372.5	0	1	n	y	0.045757	14	14	0		y	n
1055	control	80	M	0.355	133.5	1	2	y	n	0	16	16	0		y	n
1056	control	81	M	0.35	345	0	2	y	n	0.09691	14	14	0		y	n
1059	POAG	55	F	0.44	271	0	1	n	n	-0.07918	13	14	3	-4.17	y	n
1060	POAG	70	F	0.305	266.5	0	2	n	n	0.30103	17	17	2	-12.94	y	n
1061	EXG	80	F	0.485	256.5	0	1	n	n	0.39794	9	9	1	-6.57	y	n
1062	EXG	77	F	0.49	292	0	2	y	n	0.045757	14	21	3	-14.96	IOL	y
1063	POAG	72	M	0.475	280	0	2	n	n	0	31	31	4	-8.1	IOL	y
1065	control	73	F	0.31	525.5	0	2			0			0		y	n
1068	POAG	69	M	0.48	302.5	0	2	n	n	0	15	15	4	-15.52	y	n
1072	POAG	71	M	0.495	236.5	1	1			-0.07918	11	22	2	-19.97	y	n
1075	EXG	66	M	0.44	235	0	2	y	n	-0.07918	15	24	1	-0.6	IOL	y
1086	POAG	60	M	0.465	276	0	3	y	n	-0.07918	20	20	4	-4.17	y	n
1087	POAG	59	M	0.455	216	0	2	n	n	0.30103	13	23	3	-9.79	IOL	y
1091	EXG	66	M	0.295	200.5	1	0	y	n	0.823909	32	30	4	-9.21	IOL	y
1092	POAG	68	F	0.43	288.5	0	2	n	n	0.69897	13	13	5	-17.28	IOL	y
1093	EXG	70	M	0.47	290	0	2	y	n	0.09691	13	25	4	-26.02	IOL	y
1094	POAG	64	F	0.345	292.5	1	1	n	n	-0.07918	16	16	2	-6.98	IOL	y
1101	control	71	M	0.28	579.5	0	3	n	n	-0.07918	15	15	0		y	n
1102	control	83	F	0.53	242.5	0	2			0.522879			0		IOL	y
1104	POAG	75	M	0.335	404	0	3			-0.07918	16	27	3	-4.85	IOL	y
1105	POAG	73	M	0.49	249	0	2			0.221849	8	27	2	-28.75	IOL	y
1106	POAG	73	F	0.326667	347.5	0	3			0	9	46	3	-4.75	IOL	y
1107	control	61	M	0.43	337	0	3			-0.07918	14	14	0		y	n
1108	EXG	70	M	0.416667	162	0	1	y		0.522879	10	25	0	-30.54	IOL	y
1109	control	79	M	0.46	513	0	3	n	n	-0.07918	12	12	0		IOL	y
1110	control	79	F	0.42	323	0	2	y	n	0.522879	14	14	0		y	n
1113	control	71	M	0.445	237	0	2	y	n	0.045757	17	17	0		y	n
1119	POAG	62	M	0.455	267	0	3	y	n	0.90309	9	12	1	-22.68	y	y
1124	POAG	68	M	0.77	339.5	0	3	y	n	0.30103	25	25	4	-30.03	y	n
1125	POAG	34	F	0.34	263.5	1	3	n	n	-0.07918	13	13	4	-28.44	y	n

1126	POAG	64	F	0.37	562.5	0	2			0.045757	15	17	4	-12.31	y	y
1128	control	27	M	0.42	351.5	0	2	n	n	-0.07918	19	19	0	-3	y	n
1130	POAG	70	F	0.465	169	0	1			2	10	19	4	-33.95	IOL	y
1131	POAG	69	M	0.375	218	0	2	y	n	0.69897	15	15	5	-20.19	IOL	y
1135	control	75	M	0.555	294.5	0	2	n	y	0.09691	20	20	0		y	n
1136	control	68	F	0.365	291.5	0	1			-0.07918			0		IOL	y
1139	EXG	70	F	0.445	239	0	3			-0.07918	11	21	3	0.89	y	n
1141	EXG	71	M	0.34	267	1	1			0.045757	7	29	3	-26.63	IOL	y
1142	POAG	70	F	0.35	414	0	2	n	n	-0.07918	11	13	3	-27.23	IOL	y
1143	POAG	61	F	0.41	230	0	2	n	n	-0.07918	16	19	3	-8.42	y	y
1144	EXG	85	F	0.46	252	0	1			0.30103	12	27	3	-28.16	IOL	y
1146	control	50	F	0.445	663	0	3	n	n	-0.07918		12	0		IOL	y
1152	control	68	M	0.425	230	0	2	n	n	0	12	12	0		y	n
1154	EXG	78	M	0.55	405	0	2	y	n	0.221849	18	18	2	-3.66	y	n
1155	control	70	F	0.416667	215.5	0	2			-0.07918	19	20	0		y	n
1158	control	38	M	0.425	298.6667	0	1	n	n	-0.07918	12	16	0		y	n
1161	POAG	57	M	0.426667	353	0	2			-0.07918	11	19	3	-17.06	IOL	y
1162	POAG	55	F	0.415	487	0	2	n	n	-0.07918	15	20	2	-9.85	y	n
1163	POAG	81	F	0.4	398.5	0	1	n	n	0.154902	19	19	4	-24.45	y	n
1165	EXG	72	M	0.52	326.5	0	2	y	n	0	17	17	0	-0.61	y	n
1177	POAG	62	M	0.5	355.5	0	2			-0.07918	17	25	3	-19.29	IOL	y
1179	EXG	74	F	0.42	380	0	2	y	y	0	18	39	3	-19.39	IOL	y
1194	control	83	F	0.46	483	0	2	y	n	0.221849			0		y	n
1200	POAG	48	F	0.425	449.5	0	2			-0.07918	11	22	4	-20.27	y	n
1202	POAG	83	F	0.42	267	0	2	y	n	0.39794	11	13	5	-27.36	IOL	y
1204	POAG	66	M	0.395	253.5	0	3	y	n	1.39794	21	21	1	-28	IOL	y
1205	POAG	78	F	0.425	224.5	0	2			0.522879	25	32	4	-30.36	IOL	y
1206	POAG	71	M	0.385	320	0	3	n	n	0.154902	55	55	5	-27.93	IOL	y
1208	POAG	62	M	0.386667	373.5	0	1	y		0.09691	13	19	2	-24.38	y	y
1209	control	72	M	0.515	590	0	3			0.221849			0		y	n
1217	POAG	62	F	0.48	309.5	0	2	n	n	0	12	17	3	-22.59	IOL	y
1218	EXG	67	M	0.516667	345	0	2			-0.07918	13	45	3	-18.22	IOL	y
1219	POAG	72	F	0.365	519	0	3		y	0.30103	13	23	4	-24.88	IOL	y
1220	POAG	54	M	0.525	341	0	2	n	n	-0.07918	28	28	4	-20.9	y	n
1232	control	78	M	0.3	194	1	2			-0.07918	17	35	0		IOL	y
1237	control	80	F	0.37	603	0	1			0.09691			0		y	n
1238	POAG	63	M	0.465	273.6667	0	1	y	n	0.522879	23	23	4	-16.82	y	n
1239	EXG	73	M	0.485	236.5	0	1	y	n	0.39794	27	27	4	-30.45	y	n
1241	POAG	65	F	0.565	356.5	0	2			0.221849	8	8	0	-26.87	IOL	y
1242	POAG	70	M	0.46	234	0	1			-0.07918	11	16	3	-10.1	y	n
1243	EXG	80	M	0.61	207.5	0	0	y	n	0	34	36	3	-10.16	IOL	y
1253	control	80	F	0.445	349.5	0	1	n	n	0.30103	15	15	0		y	n
1255	EXG	64	M	0.46	287	0	3	y	n	-0.07918	27	27	0	-2.37	y	n
1256	POAG	65	M	0.5	368	0	2	y	n	-0.07918	25	25	3	-9.63	y	n
1263	control	72	F	0.345	414.5	0	2	y	n	0.30103	14	14	0		y	n
1275	POAG	67	M	0.46	353.5	0	3	y	n	1	16	16	3	-23.34	y	n
1276	POAG	77	F	0.53	275	0	1		n	-0.07918	19	19	2	-8.96	IOL	y
1280	EXG	68	M	0.545	196	0	2	y		-0.07918	19	29	0	-1.02	y	y
1281	control	91	F	0.335	413.5	0	3	n	n	0.221849			0		y	n
1285	EXG	67	F	0.49	357.5	0	1	y	n	-0.07918	17	22	2	-7.56	IOL	y
1286	POAG	73	M	0.46	212	1	1	y	n	-0.07918	16	21	3	-14.75	IOL	y
1292	control	84	F	0.27	812	0	2	y	n	0.045757	14	14	0		IOL	y
1293	EXG	81	M	0.423333	354.5	0	2	n	y	0.39794	15	15	0	-11.2	y	n
1294	control	68	F	0.46	384.5	0	2			-0.07918			0		IOL	y
1297	POAG	79	M	0.45	302	0	3			0.045757	11	20	3	-11.1	IOL	y
1300	POAG	73	F	0.425	245	0	2	y		0	9	27	0	-8.64	y	n
1305	POAG	83	F	0.55	318	0	2	y		1.522879	13	13	0	-16.21	y	n
1309	control	53	F	0.353333	274.5	0	2			-0.07918			0		y	n
1310	control	74	M	0.495	324	0	2	y	n	0.09691			0		y	n
1311	EXG	74	F	0.616667	267.5	0	2			0.09691	11	13	4	-31.02	IOL	y
1327	POAG	61	F	0.5	429	0	2	y	n	0.522879	15	17	2	-15.97	y	n
1329	POAG	72	M	0.475	280	0	2	n	n	0	31	31	4	-8.1	IOL	y
1332	control	68	M	0.585	389.3333	0	0			0.09691			0		y	n
1336	EXG	62	F	0.42	285.5	0	2	n	n	0.154902	21	32	3	-23.71	IOL	y
1337	EXG	69	M	0.28	321.5	1	1		y	0.154902	15	41	3	-28.04	IOL	y
1338	EXG	69	F	0.63	330	0	3	n	n	-0.07918	15	20	3	-12.92	IOL	y
1348	EXG	72	F	0.38	510.5	0	2	n	n	0.09691	27	27	3	-11.53	y	n
1349	EXG	54	F	0.395	467	0	2	n	n	0.30103	32	50	5	-22	y	n
1362	POAG	54	M	0.37	274.5	0	3	y	n	-0.07918	17	19	3	-8.23	y	y
1364	POAG	66	F	0.44	389.5	0	2	y	n	0	15	19	3	-28.36	y	y
1366	EXG	69	M	0.41	313.5	0	2	n	n	0.154902	17	17	4	-11.01	y	n
1372	control	65	M	0.395	167.5	1	1	y	n	0.30103	18	18	0		y	n
1384	control	90	F	0.43	259	0	3			0.69897			0		y	n
1385	control	83	M	0.61	296	0	2	n	n	0.39794			0		y	n
1387	control	75	F	0.553333	221	0	2			0.221849			0		y	n
1391	control	73	F	0.405	326.5	0	2	n	n	0.09691	17	17	0		y	n
1400	control	92	F	0.5	123.5	0	3	n	n	0.522879	14	14	0		y	n
1410	POAG	85	F	0.42	580	0	3			0.045757	12	12	1	-16.74	IOL	y
1411	POAG	83	M	0.53	111	0	1			0.154902	15	15	1	-27.97	IOL	y
1412	POAG	55	M	0.345	267.5	0	1	y	n	-0.07918	49	49	6	-3.48	y	n
1420	POAG	62	M	0.33	169	0	1	n	n	0.69897	52	52	0	-12.78	y	n
1425	POAG	81	F	0.5	477.5	0	2		y	1.09691	15	15	3	-6.64	y	n
